# A Hierarchical Bayesian Model for the Identification of PET Markers Associated to the Prediction of Surgical Outcome after Anterior Temporal Lobe Resection

**DOI:** 10.3389/fnins.2017.00669

**Published:** 2017-12-05

**Authors:** Sharon Chiang, Michele Guindani, Hsiang J. Yeh, Sandra Dewar, Zulfi Haneef, John M. Stern, Marina Vannucci

**Affiliations:** ^1^Department of Statistics, Rice University, Houston, TX, United States; ^2^School of Medicine, Baylor College of Medicine, Houston, TX, United States; ^3^Department of Statistics, University of California, Irvine, Irvine, CA, United States; ^4^Department of Neurology, University of California, Los Angeles, Los Angeles, CA, United States; ^5^Department of Neurology, Baylor College of Medicine, Houston, TX, United States

**Keywords:** Bayesian hierarchical model, positron emission tomography (PET), spatially-informed prior, mixture model, variable selection, Pólya-Gamma distribution

## Abstract

We develop an integrative Bayesian predictive modeling framework that identifies individual pathological brain states based on the selection of fluoro-deoxyglucose positron emission tomography (PET) imaging biomarkers and evaluates the association of those states with a clinical outcome. We consider data from a study on temporal lobe epilepsy (TLE) patients who subsequently underwent anterior temporal lobe resection. Our modeling framework looks at the observed profiles of regional glucose metabolism in PET as the phenotypic manifestation of a latent individual pathologic state, which is assumed to vary across the population. The modeling strategy we adopt allows the identification of patient subgroups characterized by latent pathologies differentially associated to the clinical outcome of interest. It also identifies imaging biomarkers characterizing the pathological states of the subjects. In the data application, we identify a subgroup of TLE patients at high risk for post-surgical seizure recurrence after anterior temporal lobe resection, together with a set of discriminatory brain regions that can be used to distinguish the latent subgroups. We show that the proposed method achieves high cross-validated accuracy in predicting post-surgical seizure recurrence.

## 1. Introduction

In the era of precision medicine, in order to deliver targeted therapies for neurological disorders, the development of methods to identify reliable and quantifiable biomarkers that are associated to individual clinical outcomes has become of paramount importance (Insel and Cuthbert, 2015). Temporal lobe epilepsy (TLE) is the most common form of adult epilepsy and the most common epilepsy refractory to anti-epileptic drugs. Surgery provides an effective treatment for many patients, yielding a seven-fold greater probability of seizure freedom 1 year after surgery than patients treated with medications alone (Wiebe et al., 2001). Despite its effectiveness, 30–50% of patients with TLE continue to experience seizures after surgery (Spencer et al., 2005; de Tisi et al., 2011).

As interictal ^18^F-fluorodeoxyglucose positron emission tomography (FDG-PET) has traditionally been used for seizure focus localization (Wieser, 2004), there is substantial interest in identifying methods that utilize PET for prediction of post-surgical seizure relief (Willmann et al., 2007). Mesial TLE with hippocampal sclerosis is defined by the presence of neuronal cell loss and gliosis in the CA1 region and endfolium of the hippocampus, a particular part of the temporal lobe (Wieser, 2004). Therefore, prediction of post-surgical outcome using FDG-PET has traditionally focused on specific regions selected a priori within the temporal lobe (Dupont et al., 2000; Lin et al., 2007). Such studies have demonstrated predictive value of FDG-PET for identifying mesial TLE. Increasing evidence, however, points at TLE as a network disorder that includes abnormality distributed beyond the temporal lobe, rather than a focal disorder (Bonilha et al., 2005; McDonald et al., 2008; Mueller et al., 2010; Chiang and Haneef, 2014). This suggests that whole-brain statistical approaches may allow for improved identification of quantifiable features from neuroimaging data that can be reliably associated with individual clinical outcomes and improve clinical decision-making.

Traditional predictive modeling approaches for neuroimaging data have included the use of pattern recognition techniques, such as Linear Discriminant Analysis (Haynes and Rees, 2005), Support Vector Machines (Mitchell et al., 2004; LaConte et al., 2005) and Bayesian classifiers (Burge et al., 2009; Arribas et al., 2010). In particular, pattern recognition techniques have been used with varying success to predict post-surgical outcome in TLE, ranging from 50 to 75% accuracy using random forests (Njiwa et al., 2015) to 70% accuracy using elastic net and support vector machines (Munsell et al., 2015). Recently, Bayesian spatial hierachical models have also been used to improve prediction accuracy from PET data by borrowing strength from spatial correlations between neighboring voxels/regions (Derado et al., 2013). Several approaches for dynamic PET data have also been proposed. O'Sullivan (2006) and Jiang and Ogden (2008), for example, utilize mixture modeling and conditional autoregressive models to incorporate spatial information into PET analysis, while other work has used functional principal components (Jiang et al., 2009) or wavelets (Millet et al., 2000; Alpert et al., 2006) to analyze dynamic PET signal. Although each of these approaches represents an important advance in neuroimaging methods development, these methods do not quantify the relative importance of selected regions, which may impact the effectiveness of related clinical decisions. Recently, Bayesian scalar-on-image regression methods have been proposed that associate a univariate outcome to massive multi-dimensional image predictors, particularly for functional magnetic resonance imaging (fMRI) data (van Gerven et al., 2010; Goldsmith et al., 2014; Li et al., 2015). All the methods above, however, do not consider the heterogeneity of the population of individuals and implicitly assume that, given a set of discriminatory regions, their association to the outcome is the same across the population. In reality, however, the strength of the association can vary across subgroups of subjects.

In this paper, we develop a statistical model to identify whole-brain biomarkers from PET imaging which are associated to the prediction of post-surgical seizure recurrence following anterior temporal lobe resection. Post-surgical seizure recurrence is thought to be due to incomplete resection of the epileptogenic zone, which is defined as the area of cortex necessary and sufficient for initiating seizures, and whose removal is necessary for seizure abolition (Lüders et al., 1993). While the epileptogenic zone was historically thought to arise from discrete focal sources, more recent evidence suggests that seizure activity arises from the activity of epileptogenic cortical networks that are distributed beyond the temporal lobe (Franaszczuk et al., 1994; Franaszczuk and Bergey, 1998; Baccalá et al., 2004; Worrell et al., 2004, 2008; Jirsch et al., 2006; Kramer et al., 2008; Chiang et al., 2017a). Patients with different epileptogenic zone configurations are expected to exhibit different likelihoods of post-surgical seizure recurrence. Different epileptogenic zone configurations are also expected to produce different interictal metabolic patterns of FDG uptake, due to the effect of epileptogenic activity on neuronal loss and postictal metabolic depression (Luders, 2008). The epileptogenic zone, however, cannot be identified pre-operatively, due to the fact that parts of an epileptogenic lesion may not be implicated in the preoperatively recorded seizure, but will continue to generate seizures post-operatively if not resected (Rosenow and Lüders, 2001). In our model formulation, we look at the observed PET brain measurements as the phenotypic manifestation of latent individual pathological states that are assumed to vary across the population. We then factor the joint distribution of the data into the product of two conditionally independent submodels, an *outcome model* that relates the post-surgical outcome to the latent states, and a *measurement model* that relates those latent states to the observed brain measurements. For the latter, we employ mixture models for clustering and variable selection priors that capture spatial correlation among neighboring brain regions. This allows us to cluster subjects into subgroups with different latent pathological states, while simultaneously identifying discriminatory brain regions that characterize the subgroups. A logistic regression model relates the latent states to the binary clinical outcome.

We apply the proposed approach to PET data collected at the University of California, Los Angeles (UCLA) as part of a clinical study on post-surgical outcomes in temporal lobe epilepsy. We also incorporate into the analysis connectivity information from resting-state functional magnetic resonance imaging (fMRI) data, to inform the selection of discriminatory brain regions. Integrative models that take into account neuroscientific information from multi-modal data sources, such as fMRI, electroencephalography (EEG), or diffusion tensor imaging (DTI), are a pressing issue in the field, in particular given the limited number of patient samples collected in many neuroimaging experiments (Bowman et al., 2012; Hinne et al., 2014; Jorge et al., 2014). Bayesian inference provides a powerful way to incorporate multi-modal imaging into computational anatomy by inclusion through network priors. In our case study, we identify a subgroup of patients at high risk for post-surgical seizure recurrence, together with several discriminatory brain regions which can be used in clinical decisions to maximize interventional treatments. Furthermore, we show that the proposed approach achieves high cross-validated accuracy in predicting post-surgical seizure recurrence. Further assessment of the performance of our method is performed in the Supplementary Material by conducting a comparison study on synthetic data against multi-step approaches and/or approaches that do not condition on latent states.

## 2. Materials and methods

### 2.1. Case study on temporal lobe epilepsy

Positron emission tomography (PET) is a type of *in vivo* nuclear medicine imaging which uses radioactive tracers to quantify tissue function. The subject is injected with a positron-emitting isotope, such as ^18^F-FDG, and a PET image is reconstructed of the isotope concentration based on the incidence of gamma rays from the positron-electron annihilation. In this work, we analyze data on 19 adult patients with drug resistant MTLE and radiological evidence of unilateral hippocampal sclerosis (MTLE-HS), who underwent pre-operative interictal ^18^F-FDG PET and anterior temporal lobe resection (ATL) at the UCLA Seizure Disorder Center between 2007 and 2012. Patients were identified from the UCLA video-EEG Epilepsy Monitoring Unit. As the primary outcome of this study was post-operative seizure freedom after epilepsy surgery, a healthy control group was not obtained as anterior temporal lobe resections are not performed in healthy patients without indication for surgery. Diagnostic evaluation included video-EEG monitoring, high resolution MRI, interictal ^18^F-FDG PET, and neuropsychological testing. PET/CT scans were acquired on a Siemens Biograph scanner as described in Kerr et al. (2013). Patients fasted for at least 6 h before each scan except for water and medications. Patients received 0.14 mCi/kg of ^18^F-FDG intravenously and rested in a quiet, dimly lit room with their eyes open during the ensuing 40 min uptake period with concomitant EEG monitoring to confirm interictal status. The iterative reconstruction program Ordered Subset Expectation Maximization (OSEM) available through NeuroQ (Syntermed, GA, USA) was used for reconstruction of PET images. Iterative reconstruction was halted after two iterations using eight subsets. CT images were reconstructed using filtered back projection at 3.4 mm axial intervals to match the slice separation of the PET data, and used for attenuation correction. Post-operative seizure freedom was assessed 1 year after surgery and classified as either seizure-free (SF; Engel Class 1) or not seizure-free (NSF; Engel Class 2–4). The binary outcome of complete freedom from disabling seizures (Engel Class 1) is the standard primary outcome of interest evaluated in epilepsy surgery treatment trials (Engel et al., 2012). The use of this primary outcome in epilepsy surgery trials results from the goal of epilepsy surgery, which is complete seizure freedom. In addition, we have available resting state fMRI (rs-fMRI) data collected on a separate set of 32 TLE patients recruited from the UCLA Seizure Disorder Center. Details on fMRI data are described in section 3.1.

### 2.2. PET pre-processing

In PET studies, the quantity that is clinically assessed is a scalar rate of regional glucose uptake, based on a method described by Sokoloff et al. (1977). This quantity is then normalized relative to an internal reference standard, such as the whole-brain or cerebellar activity, and compared to the expected level for a reference normal subject (Silverman et al., 2008). The cerebellum is commonly used as the reference PET region for diseases of interest in which the cerebellum is thought not to be affected, such as diseases involving diffuse forebrain involvement. However, cerebellar atrophy is a very well described phenomenon in epilepsy, and is moreover associated with longer duration of epilepsy as well as younger age of epilepsy onset (Sandok et al., 2000). Given that the cerebellum could be more involved in epilepsy than traditionally thought (Fountas et al., 2010), we chose to normalize by the average whole-brain uptake rather than by the cerebellum. The assessed quantity therefore provides a measure of the level of metabolic activity in each region, relative to that expected in healthy controls. Uptake levels may be quantified on the single-voxel level or based on the mean uptake within fixed regions of interest. However, because single-voxel measurements are adversely affected by noise, the use of regions of interest (ROIs) in FDG-PET has been suggested as a more robust alternative for clinical practice (Wahl et al., 2009), which additionally facilitates standardized comparisons of affected regions across subjects. NeuroQ (Syntermed, GA, USA) is a software approved by the FDA in 2004 for quantitative assessment of brain PET imaging in clinical practice and was used to pre-process PET images. Following transformation into template Montreal Neurological Institute (MNI) space by a method previously described by Tai et al. (1997), images were segmented into 47 predefined regions of interest using a predefined NeuroQ atlas (Silverman and Melega, 2004; Ercoli et al., 2012) which has been previously considered for quantitative assessment of PET data in clinical practice (Smith et al., 2007; McCallum et al., 2010; Torosyan and Silverman, 2012; Kerr et al., 2013; Akdemir et al., 2014). ROI abbreviations are listed in the Supplementary Material. Pre-processing consisted of scalp removal, rigid registration to a reference PET image to correct for head tilt, and reformatting of transaxial slices to fit normal template transaxial slices using 10 iterations. Maximization of the mutual information between the image volumes was used to identify the registration parameter. A mean count was calculated in each ROI, normalized by the whole-brain counts and standardized relative to the mean and standard deviation of each ROI among healthy controls. Greater magnitude of PET image intensities indicate more pathological levels of metabolic activity, with positive values indicating greater levels of hypermetabolism (i.e., greater metabolism than in healthy controls) and negative values indicating greater levels of hypometabolism (i.e., lower metabolism than in healthy controls). Consequently, different patterns of nonzero signal characterize different pathological patterns of metabolic activity. Imaging patterns of hyper- and hypometabolism were of interest in this study rather than the raw PET signal intensities, due to the association of hypermetabolic activity with epileptic activity. Lateralized ROIs were recoded from left and right to ipsilateral or contralateral with respect to the side of subsequent resection. A histogram of the normalized and standardized PET image intensities (Figure not shown) indicated a bell-shaped, unimodal, and fairly symmetrical distribution, with a skewness of −0.39.

### 2.3. Statistical model

Let ***X***_*i*_ denote the *R* × 1 vector of normalized PET image predictors on *R* brain regions of interest (ROIs) for subject *i* and let *Y*_*i*_ denote the corresponding post-surgical outcome, for *i* = 1, …, *n*. We propose to study the association between the PET image predictors and the outcome via a *measurement error* model formulation. As described above, non-zero values of ***X*** indicate the level of PET metabolic activity, with different non-zero intensity patterns indicating different pathological imaging profiles. Accordingly, we assume that the brain's observed profile of metabolic activity is the manifestation of a latent (i.e., unobserved) pathological state. In epilepsy, the latent pathological state represents the configuration of metabolic activity in regions implicated in the underlying epileptogenic zone, which is in turn associated to post-surgical seizure recurrence. Here, we assume a finite number of pathological states due to the expected modular organization of the brain, which is generally decomposed into a finite number of submodules (Meunier et al., 2010). Let η_*i*_ denote the latent pathological state of subject *i*. Then, we propose to factor the joint distribution of $Z_{i}={\left\{Y_{i},X_{i}\right\}}_{i=1}^{n}$ into the product of two conditionally independent sub-models: an *outcome model* that relates the clinical outcome to the latent pathological state, and a *measurement model* that relates the latent pathological state to the observed imaging data. Therefore, we consider a non-differential measurement error model, i.e., conditionally upon the latent pathological state η_*i*_, the observed surrogate ***X***_*i*_ contains no additional information on the outcome *Y*_*i*_ (Richardson and Gilks, 1993), *f*(*Y*_*i*_|η_*i*_, ***X***_*i*_) = *f*(*Y*_*i*_|η_*i*_). This model allows us to capture the current understanding in epilepsy that failure of temporal lobe resection results most likely from incomplete resection of the epileptogenic zone (Ryvlin and Kahane, 2005). In other words, if the true epileptogenic zone were known, data contained in the PET image ***X***_*i*_ would not provide any additional information on the probability of post-operative seizure recurrence *Y*_*i*_. Thus,

(1) $$\begin{array}{l} f(Z|\eta )=\overset{n}{\underset{i=1}{\prod }}f(Y_{i}|\eta_{i})f(X_{i}|\eta_{i}), \end{array}$$

where ***η*** = (η_1_, …, η_*n*_). We specify the measurement model in Equation (1) as a mixture model with variable selection. Subgroups of patients with different epileptogenic zone configurations may be expected to exhibit different risks of post-surgical seizure recurrence. We therefore specify the outcome model in Equation (1) as a logistic regression model that relates the latent states to the binary clinical outcome. There is extensive literature on the use of measurement error models to model data in which risk factors related to the observed disease or treatment status are unknown, but where surrogate measures, which provide information on the unobserved risk factor, are recorded. A review of measurement error models may be found in Carroll et al. (2006). With respect to existing literature, our model formulation allows us to cluster subjects into subgroups with different latent pathological states, i.e., different epileptogenic zone configurations, while simultaneously identifying discriminatory brain regions. In the selection, we also capture spatial correlation among neighboring brain regions via a spatial prior, as described in section 2.3.3.

#### 2.3.1. Clustering via finite mixture models

We envision that a subject may be characterized by one of *K* possible pathological states. Let η_*i*_ denote a latent random variable that identifies the state of the *i*-th subject, *i* = 1, …, *n*. We assume that the latent individual state η_*i*_ takes values in {1, …, *K*}, where one of the states can be assumed as reference. Then, for each subject *i* we define an allocation vector **ρ**_*i*_ = (*I*(η_*i*_ = 1), …, *I*(η_*i*_ = *K* − 1)), where *I*(η_*i*_ = *k*) indicates that subject *i* has latent state *k*, i.e., *I*(η_*i*_ = *k*) = 1 if η_*i*_ = *k*, and 0 otherwise. Then, for the measurement model in Equation (1), we choose a finite mixture model that clusters the *n* subjects into *K* possible subgroups as

$$\begin{array}{l} f(X_{i}|\eta_{i},\pi ,\theta )=\sum_{k=1}^{K}\pi_{k}f(X_{i}|\theta_{k}), \end{array}$$

with η_*i*_ = *k* if subject *i* belongs to cluster *k* and *P*[η_*i*_ = *k*] = π_*k*_. The η_*i*_'s are assumed to be independent and identically distributed, so that **η** ~ Multinomial (1; π_1_, …, π_*K*_). We assume a Dirichlet prior on the mixture weights, *p*(**π**) = Dirichlet (α_1_, …, α_*K*_). We consider the case where *f*(**x**_*i*_|**θ**_*k*_) is Gaussian with parameters **θ**_*k*_ = (**μ**_*k*_, **Σ**_*k*_), so that

(2) $$\begin{array}{l} f(X_{i}|\theta_{k})=N(\mu_{k},\Sigma_{k}), \end{array}$$

with *k* = 1, .., *K*. The component-specific mean **μ**_*k*_ models the latent state specific random effect and characterizes the mean metabolic profile for subjects with latent state *k*, whereas **Σ**_*k*_ is a variance-covariance matrix that captures general relationships among regions for subjects with latent state *k*. In summary, the likelihood function for the measurement model is

$$\begin{array}{l} L(X|\eta ,\mu_{k},\Sigma_{k})=\prod_{k=1}^{K}{\left(2\pi \right)}^{-n_{k}R/2}|\Sigma_{k}{|}^{-n_{k}/2}\\ \;\times \text{ exp}\left\{-\frac{1}{2}\sum_{\left\{i:\eta_{i}=k\right\}}{\left(X_{i}-\mu_{k}\right)}^{T}\Sigma_{k}^{-1}(X_{i}-\mu_{k})\right\}, \end{array}$$

with *n*_*k*_ denoting the number of subjects in cluster *k*. Here we assume diagonal variance-covariance matrices Σ_*k*_ = diag(σ_*k*,1_, …, σ_*k,R*_). Even though we make this simplifying assumption at this stage of the hierarchy, our proposed model is still able to capture structural dependencies via the specification of the prior model for the mean components in Equation (4) that we describe in section 2.3.3.

#### 2.3.2. Association with the treatment outcome

The outcome model in Equation (1) allows the prediction of the subject-specific outcomes based on the patients' individual latent pathological state η_*i*_. We can relate the latent states with the outcome of interest by employing a generalized linear model. In general, we may have available a vector of baseline covariates **U**_*i*_ for subject *i*. Since the post-surgical outcome is binary, we can then use a logistic regression model

(3) $$\begin{array}{l} p(Y_{i}=y_{i}|\eta_{i},\beta )=\frac{\text{exp}{\left(\xi_{i}^{T}\beta \right)}^{y_{i}}}{1+\text{exp}(\xi_{i}^{T}\beta )}, \end{array}$$

with **β** = (β_0_, …, β_*K*−1_, **β**_*U*_) and **ξ**_*i*_ = (1, **ρ**_*i*_, **U**_*i*_), where **β**_*U*_ is the vector of corresponding regression coefficients for $\text{U}={\left\{{\text{U}}_{i}\right\}}_{i=1}^{n}$. Here, β_*k*_, *k* = 1, …, *K* − 1 captures the “risk” associated to latent state *k* relative to the baseline latent state. Each β_*k*_ can be interpreted as the log-odds of the outcome for subjects in state *k* relative to subjects in the reference state, and β_0_ as an intercept term yielding the log-odds of the outcome for subjects in the reference state.

The analytically intractable form of the likelihood function using a logit link is known to pose challenges for Bayesian inference in logistic regression models. To address this and to improve posterior sampling, we employ the data augmentation approach recently devised by Polson et al. (2013). Let ω be a Pólya-Gamma random variable, ω ~ PG(*b, c*), with parameters *b* > 0 and *c* ∈ ℝ,

$$\begin{array}{l} \omega \overset{D}{=}\frac{1}{2\pi^{2}}\sum_{k=1}^{\infty }\frac{g_{k}}{{\left(k-1/2\right)}^{2}+c^{2}/4\pi^{2}}, \end{array}$$

where *g*_*k*_ are independently distributed as Gamma(*b*, 1). Augmentation with a Pólya-Gamma random variable allows for the likelihood contribution of the *i*th observation to be written as

$$\begin{array}{l} L_{i}(\beta )=\frac{\text{exp}{\left(\xi_{i}^{T}\beta \right)}^{y_{i}}}{1+\text{exp}(\xi_{i}^{T}\beta )}\\ \;=\frac{1}{2}\text{exp}(\kappa_{i}\xi_{i}^{T}\beta )\int_{0}^{\infty }\text{exp}\left(-\frac{\omega_{i}{\left(\xi_{i}^{T}\beta \right)}^{2}}{2}\right)\;p(\omega_{i})\partial \omega_{i}, \end{array}$$

where κ_*i*_ = *y*_*i*_ − 1/2, for ω_*i*_ ~ PG(1, 0). Combining all *n* terms then gives the following convenient representation for the conditional likelihood in **β**, given **ω** and **η**:

$$\begin{array}{l} L(\beta |\eta ,\omega )\propto \text{exp }\left\{-\frac{1}{2}{\left(z-\Xi \beta \right)}^{T}\Omega (z-\Xi \beta )\right\}, \end{array}$$

where ***z*** = (κ_1_/ω_1_, …, κ_*m*_/ω_*m*_), κ_*i*_ = *y*_*i*_ − 1/2, **Ω** = diag(ω_1_, …, ω_*n*_), Ξ is the *n* × *K* matrix $\Xi =\left(\xi_{1}^{T},\ldots ,\xi_{n}^{T}\right)$, **ξ**_*i*_ = (1, ρ_*i*,1_, ρ_*i*,2_, …, ρ_*i,K*−1_), and ρ_*i,K*_ = *I*(η_*i*_ = *k*) ∀*k* = 1, …, *K* − 1. See Polson et al. (2013) for details. We complete the model by imposing a conjugate prior on **β**, *p*(**β**) = N(**m**_β_, *V*_β_), where **m**_β_ and *V*_β_ denote the prior mean and covariance, respectively.

#### 2.3.3. Spatially-informed selection prior

Not all brain regions are expected to provide information about the subgroup structure of the subjects, in which case the inclusion of non-discriminatory regions in model (Equation 2) may obscure the discovery of true groups. One way to address this issue is through variable selection for clustering. Let **γ** ∈ {0, 1}^*R*^ denote a binary vector, where γ_*j*_ = 1 if region *j* is discriminatory, and γ_*j*_ = 0 otherwise, ∀*j* = 1, …, *R*. We follow Hoff (2006) and identify discriminatory brain regions by imposing spike-and-slab priors on the random effects **μ**_*k*_ = (μ_*k*,1_, …, μ_*k,R*_). Given the spatial contiguity in neuronal glucose consumption, we allow for spatial smoothness among neighboring regions by specifying the slab portion of the prior as an intrinsic conditional autoregressive (ICAR) prior distribution (Banerjee et al., 2014). Our prior on μ_*k,j*_ can be written as

(4) $$\begin{array}{l} p(\mu_{k,j}|\gamma_{j},\mu_{k,\backslash j})=\gamma_{j}\text{N}\left(\frac{\sum_{j^{\prime }=1}^{R}S_{j,j^{\prime }}\mu_{k,j^{\prime }}}{\sum_{j^{\prime }=1}^{R}S_{j,j^{\prime }}},\frac{c_{k}}{\sum_{j^{\prime }=1}^{R}S_{j,j^{\prime }}}\right)\\ \;+(1-\gamma_{j})\delta_{0}(\mu_{k,j}), \end{array}$$

where δ_0_ denotes a spike at zero, *S* is an *R* × *R* symmetric neighborhood matrix, with $S_{j,j^{\prime }}=1$ if regions *j* and *j*′ are neighbors, and $S_{j,j^{\prime }}=0$ otherwise, and where **μ**_*k*,\*j*_ denotes all elements of **μ**_*k*_ except the *j*th element. We also impose priors on the diagonal elements of Σ_*k*_ in Equation (2) and allow for separate variances for the discriminatory and non-discriminatory regions. In particular, for the parameters corresponding to γ_*j*_ = 1, we have σ_*k,j*_ = σ_*k*_ ~ IG(*a*_*k*_, *b*_*k*_) for all *k*, while for γ_*j*_ = 0 we impose σ_*k,j*_ = σ_0_ ~ IG(*a*_0_, *b*_0_). Finally, in specifying the prior on the selection indicators, **γ**, we allow for external information on the network structure of the brain, for example on connectivity between regions, to be incorporated in the model by imposing an Ising prior of the type

(5) $$\begin{array}{l} p(\gamma )\propto \text{exp }\left\{e1_{R}^{T}\gamma +f\gamma^{T}S\gamma \right\}, \end{array}$$

with *S* denoting the neighborhood matrix. If a connection exists between two regions *j* and *j*′, then selection of one region *j* (i.e., γ_*j*_ = 1) leads to an increased probability that region *j*′ will also be selected (i.e., $\gamma_{j^{\prime }}=1$). The hyperparameter *e* ∈ (−∞, ∞) controls the sparsity of the model and represents the prior expected number of discriminatory regions. The hyperparameter *f* > 0 is a smoothing parameter which represents the prior probability of a region being discriminatory given that its neighbors are too. In particular, if a region has no neighbors, then its prior distribution reduces to an independent Bernoulli distribution with probability exp(*e*)/(1 + exp(*e*)), which is a common prior assumed in Bayesian variable selection literature in the case of independent variables.

The prior construction (Equations 4, 5) allows for sparsity while promoting spatial contiguity in the selection. The ICAR prior, in particular, ensures that each cluster's mean metabolic PET profile varies smoothly in space, as each μ_*k,j*_ is modeled to vary around the mean of its neighbors, with variance inversely scaled by the number of neighbors. Spatial prior constructions have been used extensively in neuroimaging applications, particularly with fMRI data (Smith and Fahrmeir, 2007; Zhang et al., 2014; Li et al., 2015).

#### 2.3.4. MCMC algorithm

In order to sample from the joint posterior distribution of all parameters $\left({\left\{\sigma_{k}\right\}}_{k=1}^{K},\sigma_{0},\eta ,\pi ,\;\gamma ,{\left\{\mu_{k}\right\}}_{k=1}^{K},\beta ,\omega \right)$, we employ Markov Chain Monte Carlo (MCMC) methods that combine variable selection stochastic search algorithms that use *add-delete-swap* moves (Savitsky et al., 2011) with efficient Pólya-Gamma sampling for logit models (Polson et al., 2013). We provide full details of the implementation in the Supplementary Material.

#### 2.3.5. Prediction

An important characteristic of our model formulation is that it allows for prediction of the outcome status *y*_*f*_ of a future observation ***x***_*f*_, based on the training data {***X***, ***Y***}. In the context of pre-surgical evaluation for epilepsy surgery, this allows for probabilistic, patient-specific predictive estimates of the patient's probability of surgery benefit, in order to assist with clinical decision-making. The predictive distribution is given by

(6) $$\begin{array}{l} p(y_{f}|x_{f},X,Y)=\int_{\beta }\sum_{\eta_{f}\in \{1,\ldots ,K\}}p(y_{f}|\eta_{f},\beta )p(\beta |X,Y)p(\eta_{f}|x_{f})\partial \beta , \end{array}$$

and cannot be computed in closed form. Following standard Bayesian techniques, these steps can be employed to simulate from Equation (6):

1. Sample *T* values of **μ**_*k*_, Σ_*k*_, π_*k*_, **β** from the joint posterior, using the MCMC algorithm as described in the Supplementary Material.
2. For each posterior draw, *t* = 1, …, *T*:
  - Sample *m* ≥ 1 values of η_*f*_ ∈ {1, …, *K*} from *p*(η_*f*_|***x***_*f*_), where ∀*k* = 1, …, *K*
    $$\begin{array}{l} p(\eta_{f}=k|x_{f})\propto p(x_{f}|\eta_{f}=k)p(\eta_{f}=k)=p(x_{f}|\mu_{k}^{\left(t\right)},\Sigma_{k}^{\left(t\right)})\pi_{k}^{\left(t\right)}. \end{array}$$
  - For each sampled value of η_*f*_, sample a value of *y*_*f*_ ∈ {0, 1} from $p\left(y_{f}|\eta_{f},\beta^{\left(t\right)}\right)$.

The posterior predictive probability *p*(*y*_*f*_ = 1|***x***_*f*_, ***X***, ***Y***) can then be estimated as the proportion of posterior predictive samples for which *y*_*f*_ = 1. In the analyses of this paper, given the limited number of samples available, which does not allow a meaningful splitting of the data into training and validation, we implemented cross-validation prediction via the importance-sampling approach, as proposed by Gelfand (1996), and write the cross-validation predictive density for the *i*th observation as

$$p(Y_{i}=1|X,Y_{-i})=\int_{\eta ,\beta }p(Y_{i}=1|X,Y_{-i},\eta ,\beta )p(\eta ,\beta |X,Y_{-i})\partial \beta \partial \eta$$

where we use *p*(**η, β**|***X***, ***Y***) as an importance sampling density for *p*(**η, β**|***X***, ***Y***_−*i*_), and *Y*_−*i*_ denotes the non-hold out outcomes. Specific details on implementation are provided in the Supplementary Material.

## 3. Results

We now apply the proposed model to the data we have available from the University of California, Los Angeles Seizure Disorder Center, where we illustrate the utility of our proposed model for predicting a post-surgical outcome among MTLE-HS patients from pre-surgical FDG-PET imaging.

### 3.1. Prior connectivity network

For this analysis, we allowed the spatial network prior Equation (5) to capture information on functional connectivity between the ROIs, which we estimated based on resting-state fMRI data (rs-fMRI), collected on a separate set of 32 unilateral temporal lobe epilepsy patients from the UCLA Seizure Disorder Center. Rs-fMRI was performed on the subjects after a comprehensive epilepsy surgery evaluation and prior to epilepsy surgery. None of the patients had a seizure in the 24 h preceding the imaging or had seizures during the study, as confirmed by the simultaneous EEG obtained during fMRI. There were no post-surgical outcome data available for these patients. External or historical information is often used to formulate priors in Bayesian analysis. There is extensive literature which demonstrates the general replicability of Pearson correlation estimation of functional connectivity from rs-fMRI in temporal lobe epilepsy (Centeno and Carmichael, 2014). Furthermore, despite increasing evidence that functional connectivity is dynamic (Honey et al., 2009; Ma et al., 2014; Chiang et al., 2016), recent research indicates a large proportion of the information present in functional connectivity is contained in static estimates (Chiang et al., 2017b).

**Figure 1:**
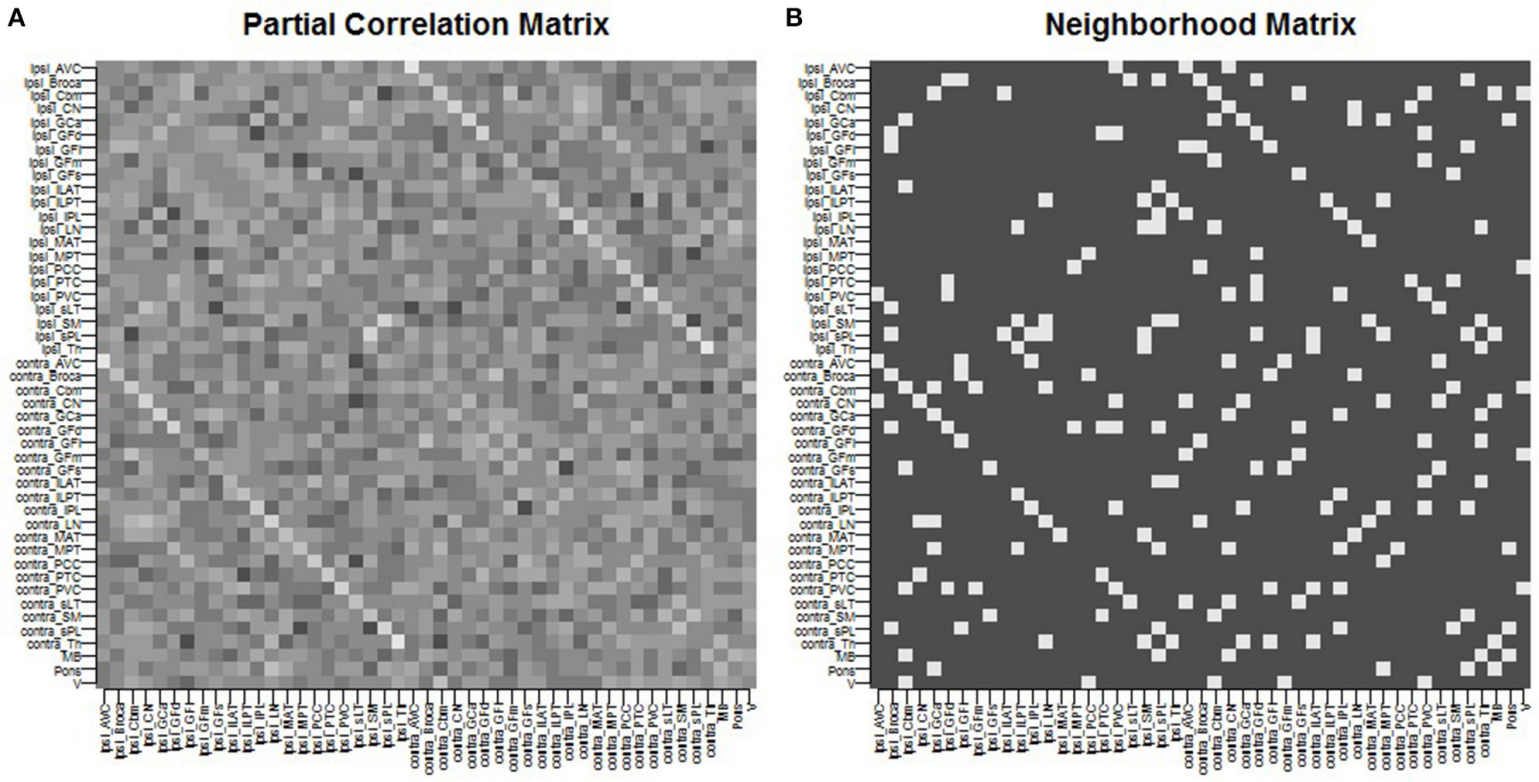
Spatial connectivity network between ROIs constructed from resting-state fMRI imaging: **(A)** partial Pearson correlation matrix, **(B)** neighborhood matrix *S* of binarized edges.

We give full details of the rs-fMRI data and the process to estimate a connectivity network in the Supplementary Material. In brief, preprocessing of rs-fMRI imaging was performed using FSL (fMRIB Software Library) version 5.0.7 (Oxford, United Kingdom, www.fmrib.ox.ac.uk/fsl). Functional connectivity between the 47 ROIs was estimated by placing a 6-mm spherical seed in Montreal Neurological Institute (MNI) space at the location of each of the 47 ROIs. Each patient's fMRI BOLD image was registered to the patient's high-resolution structural image using FLIRT (FMRIB's Linear Image Registration Tool) (Jenkinson et al., 2002; Greve and Fischl, 2009), and the high-resolution structural was registered to the standard MNI space using FNIRT (FMRIB's Non-linear Image Registration Tool) (Andersson et al., 2007). Functional connectivity between each pair of nodes was computed as the partial Pearson correlation between the averaged regional time-series. This provided us with a 47 × 47 correlation matrix. An edge was then considered as included in the connectivity network if the correlation between the regions exceeded a given threshold. The threshold was chosen so that the average number of neighbors for each region was approximately 5, yielding a connectivity structure close to a three-dimensional lattice. The resulting network was used as the neighborhood matrix *S* in the specification of the MRF prior (Equation 5) on γ and also in the ICAR prior (Equation 4) on the slab portion of the prior on μ_*k,j*_. The estimated functional connectivity matrix and resulting neighborhood matrix *S* are shown in Figure 1. We observe several known connectivity relationships, including functional connectivity between regions in the brainstem (midbrain, pons); between the primary and associative visual cortices; between the cerebellar hemispheres and vermis; and between ipsilateral and contralateral ROIs (Quigley et al., 2003).

### 3.2. Biomarker selection and clustering

In our approach to model fitting we consider a grid of values of *K* to find the number of states *K* yielding the best model fit that also provides improved clinical interpretability. For the study of this paper, model fit for each value of *K* for *K* = 2, …, 6 was assessed using the deviance information criterion (DIC) of Spiegelhalter et al. (2002). We found that *K* = 2 clusters allowed for a parsimonious model permitting meaningful clinical characterization of high- and low-risk patients, with minimal to no further improvement in the DIC for larger values of *K*. This result was confirmed through model comparison using the posterior Bayes factor (Aitkin, 1991), with a posterior Bayes factor greater than 1 from comparisons of the *K* = 2 model to *K* = 3, …, 6 models. Results we report here are based on the combined posterior output from two MCMC chains, with each chain initialized with different numbers of discriminatory ROIs and number of subjects in each subgroup. Other initial values were set as $\mu_{k}^{\left(0\right)}=$ **0**, $\sigma_{k}^{\left(0\right)}=1\;\forall k$, $\sigma_{0}^{\left(0\right)}=1$, **β**^(0)^ = **0**. We ran each MCMC chain for 100,000 iterations, with the first 50,000 sweeps discarded as burn-in.

As discussed in section 2.3.3, the hyperparameter *e* of the MRF prior (Equation 5) regulates the prior sparsity whereas *f* induces smoothness, with higher values of *f* yielding a higher prior probability that a region is selected given that its neighbors are selected. The choice of *e* and *f* has been discussed by Li and Zhang (2010) and Stingo et al. (2013). It is known that with distributions as in Equation (5) a phase transition boundary problem can be encountered, where the number of selected regions increases sharply for small changes in *f* (Li and Zhang, 2010). Here we set the sparsity parameter to *e* = −4.5, corresponding to a lower bound on the prior probability of selection of 1%. As for the prior smoothness, *f*, a plot of the prior over a grid of values *f* ∈ {0.1, 0.2, 0.3, …, 0.9} revealed that the phase transition starts at a prior smoothness of *f* = 0.2 and becomes severe at around *f* = 0.4. As suggested by Li and Zhang (2010), the prior smoothness parameter *f* was therefore set to a value far from the phase transition boundary. Here we present results for two values, *f* = 0.01 and *f* = 0.1, representing different levels of small-to-moderate effect of the prior information on connectivity. As for the other hyperparameter settings, we placed a vague prior on the mixing parameters **π**, that is, α_*k*_ = 1 ∀*k*, and fixed the prior shape and scale parameters of the inverse gamma priors on σ_*k*_ and σ_0_ to be non-informative with *a*_*k*_ = 2 and *b*_*k*_ = 1 ∀*k*, and *a*_0_ = 2 and *b*_0_ = 1. We also set the unscaled variance of the ICAR prior to *c*_*k*_ = 5, and the prior mean and covariance of **β** to ***m***_β_ = **0** and *V*_β_ = 5𝕀, respectively. Age of the patient at surgery, epilepsy duration, and history of generalized tonic clonic seizures were controlled for as baseline covariates in the logistic likelihood.

Convergence of each MCMC chain was assessed using two independent tests: the Raftery-Lewis diagnostic (Raftery and Lewis, 1992) and the Geweke test (Geweke, 1991). In addition, convergence of the multiple chains was assessed using the Gelman-Rubin potential scale reduction factor, based on the implementation in the R package “coda” (Raftery and Lewis, 1992). Convergence diagnostics indicated convergence to the stationary distribution (results reported in the Supplementary Material). Agreement between MCMC chains was assessed through the Pearson correlation between the marginal posterior probabilities of ROI selection and cluster allocation of each pair of chains.

**Figure 2:**
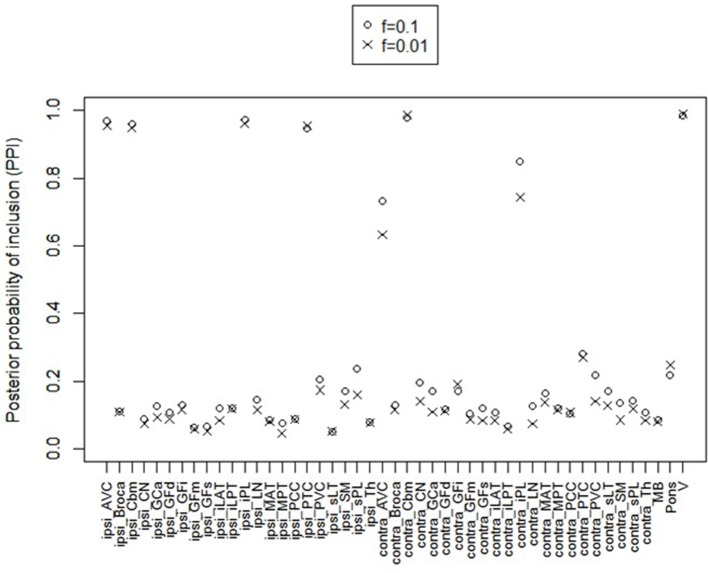
Temporal lobe epilepsy dataset: Marginal posterior probabilities of inclusion for brain regions, for *f* = 0.01 (x) and *f* = 0.1 (o).

**Figure 3:**
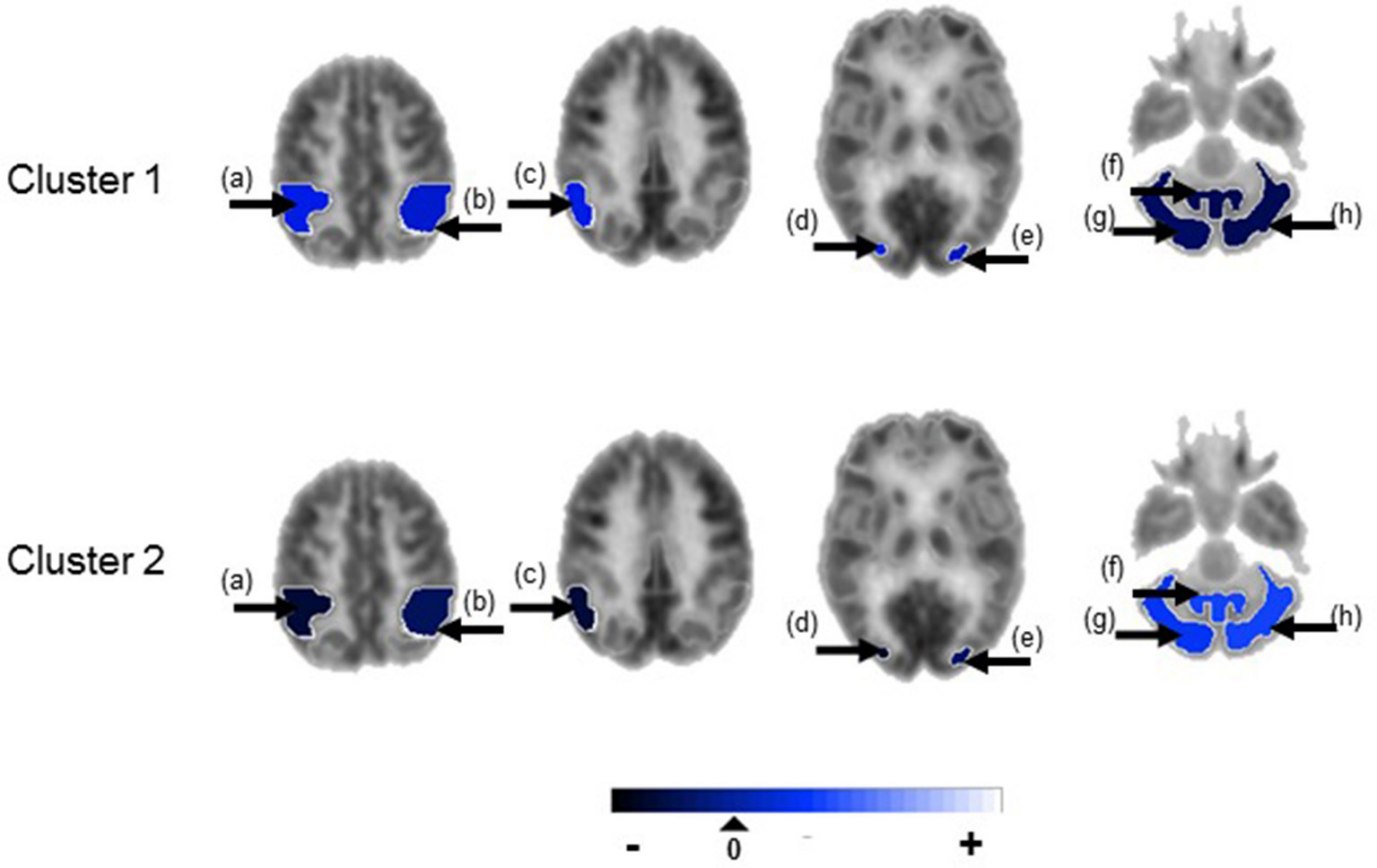
Temporal lobe epilepsy dataset: Mean random effect (**μ**_*k*_) of PET metabolic activity for ROIs with PPI greater than 0.5, shown on axial sections. (a) Ipsilateral inferior parietal lobule, (b) contralateral inferior parietal lobule, (c) ipsilateral parieto-temporal cortex, (d) ipsilateral associative visual cortex, (e) contralateral associative visual cortex, (f) cerebellar vermis, (g) ipsilateral cerebellar hemisphere, (h) contralateral cerebellar hemisphere. Non-selected ROIs are shown in grayscale.

For posterior inference, our primary interest is in the estimation of the discriminatory regions, the latent states, and their association with the binary clinical outcome, as captured by the parameters **γ**, **η**, and **β**, respectively. Trace plots for these parameters showed good mixing for all chains (figures not shown). Figure 2 shows the marginal posterior probabilities of inclusion (PPIs) for each of the 47 brain regions, with different graphical symbols for the settings of *f* = 0.01 (x) and *f* = 0.1 (o). Based on this plot, a selection of the discriminatory regions can be done by thresholding the PPIs. For example, the median model (Barbieri and Berger, 2004) selects the same subset of 8 ROIs under both *f* = 0.01 and *f* = 0.1. The selected brain regions are listed in Table 1, and graphically depicted in Figure 3. To examine the sensitivity of the selected regions to the formulation of the network prior, we additionally ran the model under a neighborhood matrix *S* defined by simple Euclidean distance. Selected discriminatory regions were robust to the formulation of the network, with the exception of the contralateral associative visual cortex, which had a marginal PPI of 0.303 (*f* = 0.1) and 0.311 (*f* = 0.01) under a network defined by spatial neighbors. This decrease in posterior probability is an effect of the MRF prior, due to the functional connectivity present between the ipsilateral and contralateral associative visual cortex in Figure 1B which is not captured based on spatial distance.

**Table 1:** Temporal lobe epilepsy dataset: Selected brain regions and corresponding marginal posterior probabilities of inclusion (PPI).

**ROI**	**PPI**	
	***f* = 0.01**	***f* = 0.1**
Ipsilateral inferior parietal lobule	0.961	0.973
Ipsilateral parietotemporal cortex	0.955	0.948
Ipsilateral associative visual cortex	0.956	0.969
Contralateral inferior parietal lobule	0.742	0.850
Contralateral associative visual cortex	0.632	0.732
Contralateral cerebellar hemisphere	0.988	0.979
Ipsilateral cerebellar hemisphere	0.950	0.961
Cerebellar vermis	0.989	0.984

**Figure 4:**
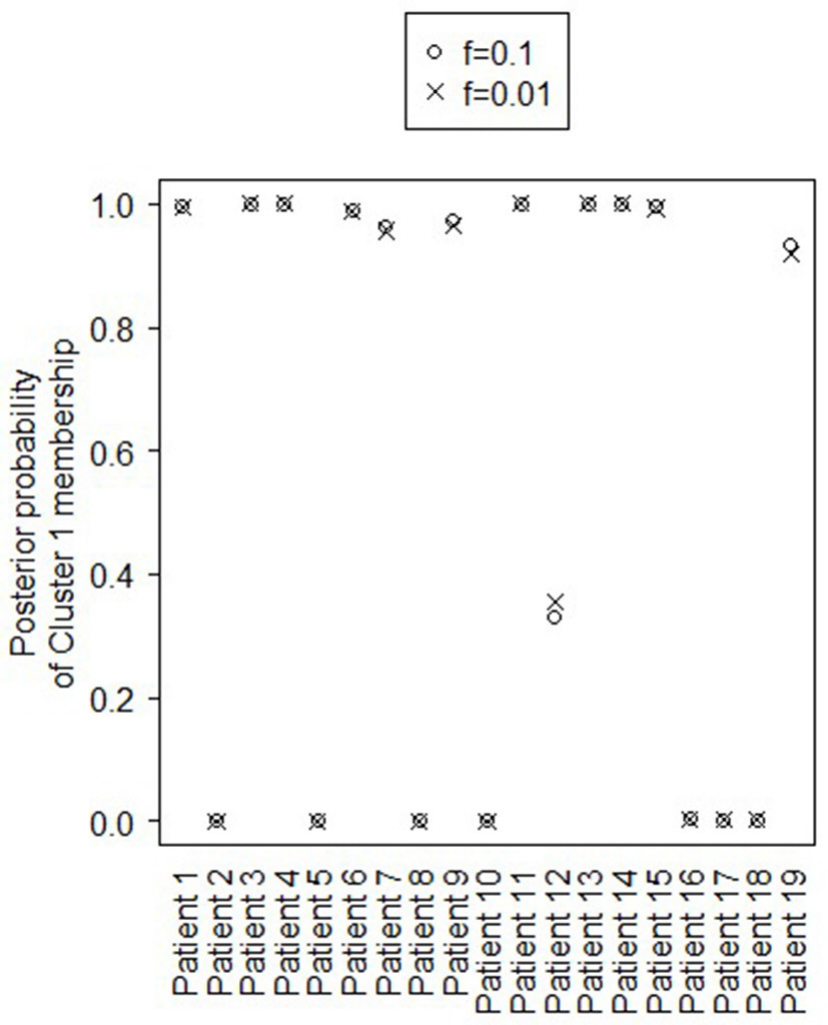
Temporal lobe epilepsy dataset: Marginal posterior probabilities of cluster allocation, for *f* = 0.01 (x) and *f* = 0.1 (o).

**Figure 5:**
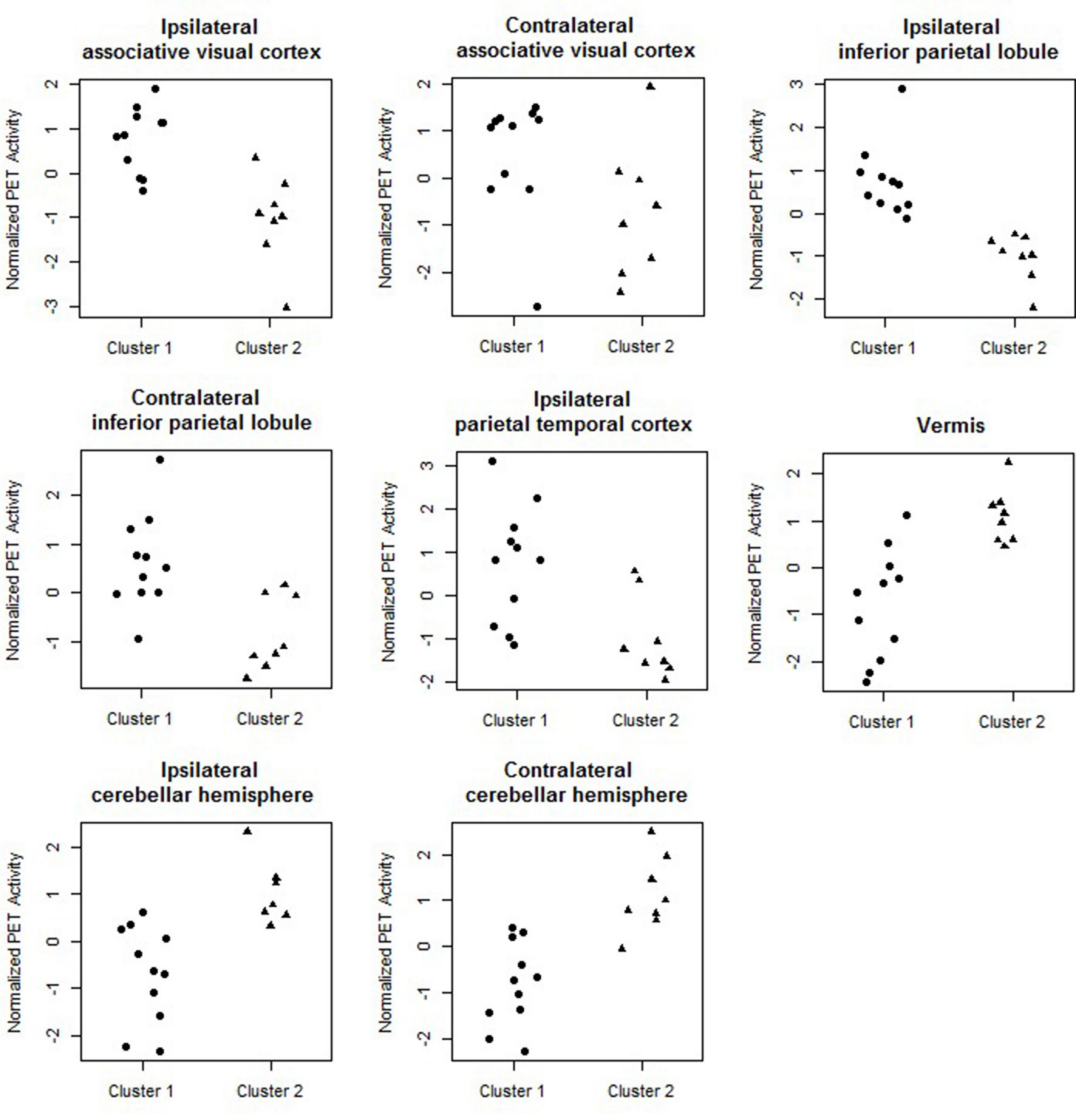
Temporal lobe epilepsy dataset: Distribution of PET metabolic activity in the selected regions for the identified subgroups, for *f* = 0.1.

Figure 4 shows the marginal posterior probabilities of sample allocations for each of the 19 MTLE-HS patients. A classification of the subjects into two subgroups can be obtained, for example, by assigning subjects according to the posterior mode of **η**. For interpretation of the two subgroups, one can examine the PET metabolic activities characterizing the subjects. These are shown in Figure 5 for the selected brain regions. Furthermore, posterior inference for the **β** parameters is summarized in Table 2. These results suggest that the two subgroups identify patients at different levels of risk for post-operative seizure recurrence, with one subgroup having a *e*^β^ = 5.2 times greater odds of persistent post-operative seizures 1 year after surgery (Table 2). This corresponds to a 90% posterior probability of an odds ratio >1 for post-surgical seizure freedom between the two identified subgroups (Table 2). Figure 5 reveals, in particular, that the subgroup with greater odds of post-operative seizure recurrence (Cluster 2) is characterized by lower levels of interictal glucose metabolism in the bilateral associative visual cortices, ipsilateral parieto-temporal cortex, and bilateral inferior parietal cortices, as well as higher levels of interictal glucose metabolism in the bilateral cerebellar hemispheres and cerebellar vermis. Our identification of these metabolic patterns may suggest extratemporal gliosis, as well as increased baseline levels of cortical excitability, in patients at higher risk for post-operative seizure recurrence. We provide further comment on the neurological significance of these findings in the Discussion.

**Table 2:** Temporal lobe epilepsy dataset: (a) Posterior mean of **β**; (b) 95% credible interval (CI) for **β**; and (c) posterior probability of odds ratio >1, e.g., $\mathbb{P}\left[e^{\beta_{j}}>1|X,Y\right]=\mathbb{P}\left[\beta_{j}>0|X,Y\right]$, shown for proposed approach (*f* = 0.1), multi-step logistic approach, and multi-step sparse clustering approach.

	**Proposed Method**			**Multistep logistic regression**			**Multistep sparse clustering**		
	**(a)**	**(b)**	**(c)**	**(a)**	**(b)**	**(c)**	**(a)**	**(b)**	**(c)**
β_0_	−0.377	(−1.588, 3.587)	0.43	−0.225	(−0.8, 1.665)	0.41	−0.169	(−0.746, 1.718)	0.43
β_1_	0.368	(0.275, 0.685)	0.99	0.250	(0.183, 0.48)	0.99	0.262	(0.196, 0.486)	0.99
β_2_	−3.726	(−4.639, −0.76)	0.01	−1.25	(−1.742, 0.347)	0.06	−1.247	(−1.737, 0.358)	0.06
β_3_	−0.082	(−0.117, 0.032)	0.09	−0.069	(−0.094, 0.011)	0.05	−0.079	(−0.107, 0.014)	0.05
β_4_	1.649	(0.832, 4.364)	0.90	0.939	(0.453, 2.552)	0.88	0.41	(−0.113, 2.140)	0.68

*Here β_1_ ≡ Epilepsy duration, β_2_ ≡ History of GTC, β_3_ ≡ Age at surgery, β_4_ ≡ Cluster 1 (v. Cluster 2)*.

*Odds are with respect to seizure freedom*.

### 3.3. Prediction results

In addition to the identification of subgroups of subjects, characterized by latent pathologic conditions differentially associated to the outcome of interest, and the selection of imaging biomarkers that characterize the pathologic states of the subjects, our modeling approach allows a probabilistic estimate of an individual patient's risk of post-operative seizure recurrence. Probabilistic assessment of outcome risk may aid pre-surgical decision-making, by facilitating identification of patients with greater probability of seizure recurrence following anterior temporal lobe resection. Such information may potentially be weighed against the known risks of surgery (e.g., infection, bleeding, reactions to general anesthesia) to stratify patients according to predicted outcome. Here, we assessed prediction performance via importance-sampling cross-validation.

**Figure 6:**
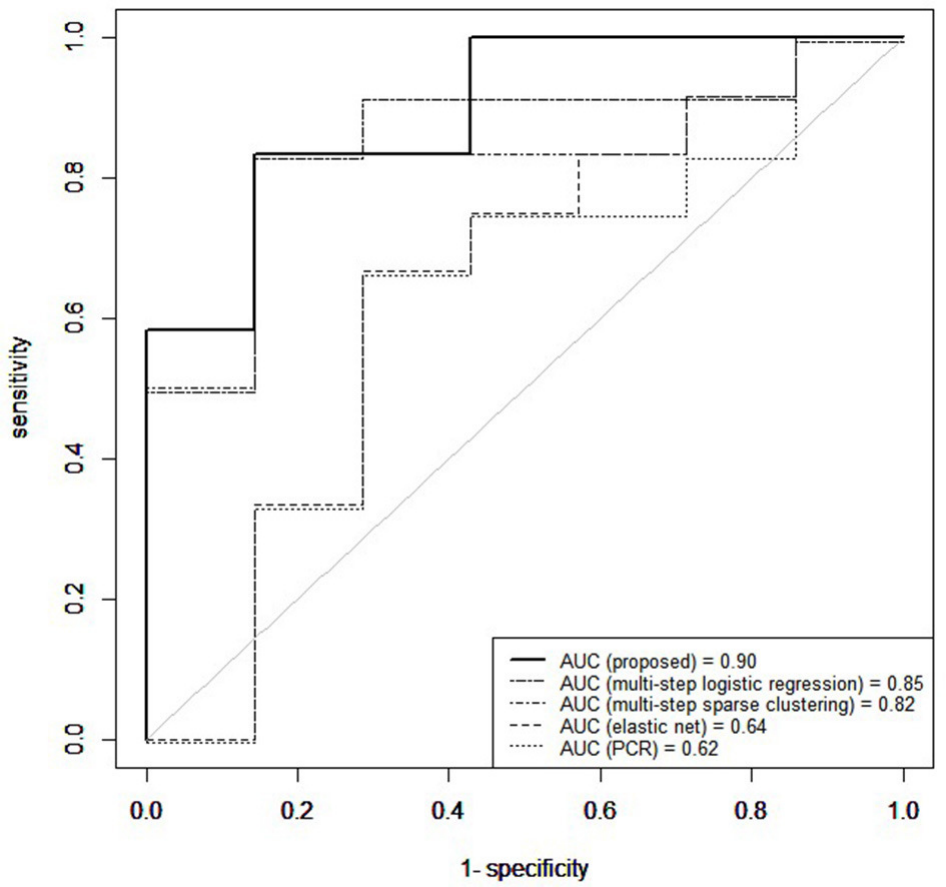
Temporal lobe epilepsy dataset: Receiver operating characteristic curve (ROC) for proposed method, elastic net, principal components regression (PCR), multi-step logistic regression, and multi-step sparse clustering in predicting post-operative outcome 1 year after anterior temporal lobe resection.

Figure 6 shows the receiver operating characteristic (ROC) curve, a plot of the false positive rates vs. the true positive rates, obtained for a grid of threshold values (0:0.05:1) on the estimated posterior predictive probabilities. The area under the curve (AUC) was 0.91. The optimal threshold, selected to maximize the Youden's index (Hiden and Glasziou, 1996), for imbalanced class sizes, resulted in an 84% predictive accuracy, with correct prediction of post-surgical outcome in 16/19 patients, including 10/12 seizure-free patients and 6/7 non seizure free patients.

Our prediction results compared favorably to those we obtained on the same data with other analogous methods which predict binary outcomes from an identified underlying latent state. In particular, we compare to three multi-step approaches commonly used in prediction for their simplicity and computational speed. In the first approach, principal components was used to reduce the data to the top eight principal components, collectively explaining 85% of the variance in the data. The reduced principal components of **X** were then used as predictors within Bayesian logistic regression. Predictive accuracy was assessed through the importance-sampling cross-validation prediction approach of Gelfand (1996). In the second approach, a multistep logistic regression approach was used, similarly to what has been done in neuroimaging studies (Versace et al., 2014). In this approach, a filtering approach was performed by calculating permutation *p*-values for each region and retaining regions with small *p*-values. Using this reduced subset of regions, patients were clustered using *k*-means. Bayesian logistic regression was fitted to predict post-surgical outcome from latent class membership, and importance sampling cross-validation used to assess predictive accuracy. In the third comparison, a multi-step version of our approach was used, in which sparse cluster analysis was separated from the outcome model. In particular, a greedy forward search algorithm was used for simultaneous variable selection and clustering (Raftery and Dean, 2006). Patients were clustered based on the selected variables through a Gaussian mixture model (Fraley et al., 2012) and Bayesian logistic regression then used to predict post-surgical outcome from latent class membership, with predictive accuracy assessed through importance-sampling cross-validation. Prediction results using our unified approach attained superior predictive performance compared to multi-step approaches (Figure 6). Multi-step logistic regression and multi-step sparse clustering approaches attained higher predictive accuracy than PCR. We also compared to methods such as elastic net (Zou and Hastie, 2005), ridge regression (Hoerl and Kennard, 1970), and the Least Absolute Shrinkage and Selection Operator (LASSO) method of Tibshirani (1996) that, in particular, do not condition on latent states, but rather use the ***X*** data as the covariates. Penalized regression approaches that did not condition on a latent state performed poorly in data with underlying latent states (see Supplementary Material). Additionally, in the Supplementary Material, we conduct a full comparison study among competing methods on synthetic data to evaluate results for both prediction and biomarker selection.

## 4. Discussion

Our results have identified a subgroup of temporal lobe epilepsy patients with 5.8 times greater odds of post-operative seizure recurrence after anterior temporal lobe resection. These patients were characterized by lower levels of interictal metabolism in regions near the ipsilateral parieto-temporal-occipital junction. Lower interictal metabolism in peritemporal regions may suggest structural abnormalities such as gliosis or neuronal loss in these regions, alternatively or in combination with functional abnormality involving a widespread epileptogenic network which extends beyond the temporal lobe. Evidence for such a subgroup has been suggested by previous research, which found limited improvement in seizure outcomes in patients with electrocorticographical (ECoG) evidence of extratemporal involvement of inferior parietal cortex (Aghakhani et al., 2004). The implication of extratemporal brain structures in patients with poorer postsurgical outcomes supports the presence of latent pathologies in patients with epilepsy. Other ECoG studies have also suggested the presence of latent pathology in epilepsy involving spread of the epileptogenic focus and the possible creation of secondary foci (Rougier, 1990; D'Ambrosio et al., 2005). Therefore, lower interictal metabolism in this subset of patients may suggest a subtype of MTLE-HS with parietal involvement, which may lead to post-operative seizure generation if not resected. The involvement of posterior parietal regions in this subset of patients may result from connectivity to other regions clinically involved in MTLE. Structural connectivity exists between the presubiculum and the posterior parietal cortex through the cingulum, for example, and functional connectivity between these regions also exists through the default mode network (Buckner et al., 2008). Pulvinar atrophy has also been found in TLE patients with persistent post-operative seizures (Keller et al., 2015), so connectivity of posterior parietal regions to the pulnivar nucleus may also play a role in posterior parietal involvement.

Patients at high risk for post-operative seizure recurrence were also characterized by higher levels of interictal glucose metabolism in the cerebellum. The cerebellum's role in inhibiting seizures has been investigated since the early 1940's, following the discovery that cerebellar stimulation may result in seizure modification or even termination (Moruzzi, 1950). Recent technological advances in techniques for cerebellar stimulation have led to renewed interest in the role of cerebellar stimulation in seizure inhibition, with a 41% seizure rate reduction achieved through cerebellar stimulation (Velasco et al., 2005). Direct optogenetic stimulation of the cerebellar Purkinje cells has been found to be sufficient to reduce the duration of seizures in temporal lobe epilepsy (Krook-Magnuson et al., 2014). It is postulated that the mechanism of cerebellar stimulation in seizure inhibition may be through increased inhibitory efferent output from the Purkinje cells to the deep cerebellar nuclei, resulting in increased inhibitory cerebellar output to the thalamocortical projections and thus decreased contralateral cortical excitability (Fountas et al., 2010). Likewise, the cerebral cortex exhibits feedback to the contralateral cerebellar hemispheres through corticopontocerebellar tracts. In our study, we found that the subgroup of MTLE-HS patients at high risk for post-operative seizure recurrence was characterized by higher levels of interictal glucose metabolism in the bilateral cerebellar hemispheres and cerebellar vermis, with slightly larger marginal posterior probability of discriminating high- vs. low-risk patients in the contralateral than the ipsilateral cerebellar hemisphere. Higher interictal glucose metabolism in the cerebellum may be caused by pre-operatively increased baseline levels of cortical excitability in high-risk patients, resulting in increased activity of corticopontocerebellar white matter tracts and increased crossed cerebellar metabolism. The localization of this phenomenon may be similar to that of cerebellar diaschisis, in which supratentorial lesions such as stroke may cause disruption of corticopontocerebellar tracts and therefore contralateral cerebellar hypometabolism. In the case of epilepsy, in which there is over- rather than underactivity of the cortex, overstimulation of the corticopontocerebellar tracts may lead to contralateral cerebellar hypermetabolism. Inhibitory outflow from the Purkinje cells may then result in hypometabolic activity in areas such as the inferior parietal lobule, congruent with the functional abnormality observed in the temporo-parieto-occipital junction as described above. Our observation of bilaterally increased glucose metabolism in the cerebellum suggest bilaterally increased cortical excitability in patients at high risk for post-operative seizure recurrence, with slightly higher cortical excitability ipsilaterally. The greater contralateral cerebellar involvement observed here is also consistent with our observation of ipsilaterally involved temporo-parieto-occipital regions due to crossed cerebello-cortical connections.

In addition to enhancing understanding of the pathophysiology behind post-operative seizure recurrence, our finding that patients at high risk for epilepsy surgery failure are characterized by lower PET metabolism in peritemporal regions and higher cerebellar metabolism, provides a marker for patients where epilepsy surgery is at high risk for failure. These patients may be better candidates for neuromodulatory treatments for medication-refractory epilepsy, such as direct cortical stimulation, as is being used in responsive neurostimulation (RNS) at regions of seizure onset (Geller et al., 2017). We show that TLE patients at high risk for anterior temporal lobe resection failure have abnormal pre-surgical brain metabolic activity compared to those patients who attain post-surgical seizure freedom, suggesting a difference in the underlying brain networks of the two groups. The approach proposed here provides a method which may potentially allow for pre-surgical differentiation between patients with abnormal underlying brain activity.

In this paper we have developed a general integrative modeling framework to characterize the association between a set of image predictors and an individual clinical outcome that simultaneously (a) identifies subgroups of patients characterized by latent pathologies differentially associated to the outcome of interest, (b) identifies discriminatory brain regions across subjects, and (c) uses prior connectivity information from external data to inform the selection of biomarkers. Our Bayesian measurement error model provides a modeling approach for the prediction of post-surgical treatment response from imaging data which explicitly accounts for the unobserved disease state. As described in section 2.3.5, our model provides an approach in which a new prospective surgery candidate can come in, be scanned with PET imaging, assigned to a latent risk group, and evaluated for their probability of achieving seizure freedom if operated upon. By accounting for heterogeneity in the unobserved state, while allowing for incorporation of external prior information, we have obtained accurate prediction in data where surrogate measures, such as neuroimaging data, are observed. We have shown that our approach achieves superior predictive performance compared to commonly used approaches, such as principal components regression, ROI-based clustering, and ROI-based sparse regression, and additionally leads to accurate inference with respect to identification of latent states and variable selection.

We have used the proposed method to analyze data we have available from the University of California, Los Angeles Seizure Disorder Center, where the interest was in predicting the post-surgical outcome among MTLE-HS patients from pre-operative FDG-PET imaging. In the analysis, we have used resting-state fMRI imaging to inform the prior model. Our analysis has identified several discriminatory ROIs, together with a subgroup of patients at higher risk of post-operative seizures recurrence. Pre-surgical identification of regions pathophysiologically involved in post-operative seizure recurrence may assist in targeting these regions for interruption. Here, patients at higher risk were characterized by lower levels of interictal glucose metabolism in the bilateral associative visual cortices, ipsilateral parietotemporal cortex, and bilateral inferior parietal lobules, and higher levels of interictal glucose metabolism in the bilateral cerebellar hemispheres and cerebellar vermis. Cross-validated prediction of post-operative seizure freedom has achieved an AUC of 0.91 and 84% predictive accuracy, showing superior predictive performance compared to methods which do not condition on latent states. One caution in interpreting the results of this study is the moderate statistical power due to limited sample size. Future corroboration on larger samples is needed prior to use in clinical practice. Pre-surgical identification of patients at high risk of not benefiting from surgery may improve treatment planning for these patients, including the potential avoidance of surgery risks in cases with low probability of benefit.

In our study, we have utilized standard PET ROIs obtained from quantitative assessment software used in clinical practice, where PET activity in each region of interest is computed by averaging within the ROI. Similar ROI-based approaches are utilized within the standard preprocessing protocol of NeuroQ to aid clinical interpretability, and have demonstrated clinical utility in neurological disorders such as Parkinson's disease (Akdemir et al., 2014), tinnitus (Smith et al., 2007), and epilepsy (Kerr et al., 2013). However, it is important to note that voxel-based data allow for a finer-grained approach to biomarker selection and may be of interest in future applications of our methodology. Use of other well-known atlases to segment PET data, such as the Automated Anatomic Labeling (AAL) atlas, may also be useful for comparing to other studies. Rigid registration and the use of PET-to-PET registration is also susceptible to PET signal variations, with hippocampal atrophy in TLE potentially contributing further to decreased registration accuracy as well as partial voluming effects. Further improvements in predictive accuracy may be seen with alternative pre-processing methods, including registration to high-resolution structural imaging and partial volume correction.

Future applications of our method to pre-operative mapping may wish to investigate finer parcellations of the brain, to better delineate the epileptogenic zone and more directly aid pre-operative mapping. Given the routine use of fMRI and EEG in the management of patients with epilepsy, it might also be possible to extend our general model formulation to the identification of spatial fMRI markers of disease outcome while taking advantage of the temporal resolution of EEG data to construct prior connectivity networks. Finally, even though the motivating example for our proposed model has come from the prediction of post-surgical outcomes in epilepsy surgery, data from other neurological disorders may also be analyzed. In such cases, it may be of interest to extend the treatment outcome to a multinomial likelihood, with larger sample sizes needed if such analysis is desired.

## Ethics statement

In this paper we analyze data that were collected as part of a clinical study between 2007 and 2012. No new data were generated for this manuscript. The data were provided to us in computer form and were recorded in such a manner that subjects could not be identified directly or indirectly through identifiers/codes. The protocol that generated the data is now closed to enrollment.

## Author contributions

SC, MG, ZH, JS, and MV contributed to the design and analysis of the work; HY, SD, and JS contributed to the data acquisition; SC, MG, and MV wrote the paper; all authors revised the manuscript critically for important intellectual content.

### Conflict of interest statement

The authors declare that the research was conducted in the absence of any commercial or financial relationships that could be construed as a potential conflict of interest.
