# Supplementary material for: A Hierarchical Bayesian Model for the Identification of PET Markers Associated to the Prediction of Surgical Outcome after Anterior Temporal Lobe Resection
Source: Front Neurosci. 2017 Dec 5;11:669. doi: 10.3389/fnins.2017.00669 (PMC5723403; doi:10.3389/fnins.2017.00669)
Supplement: Supplementary file 1 [file Presentation1.pdf]

# *Supplementary Material*: A Hierarchical Bayesian Model for the Identification of PET Markers Associated to the Prediction of Surgical Outcome After Anterior Temporal Lobe Resection

**Sharon Chiang** [1,2], **Michele Guindani** [3], **Hsiang J. Yeh** [4], **Sandra Dewar** [4], **Zulfi Haneef** [2], **John M. Stern** [4] **and Marina Vannucci** [1,*]
*Correspondence:
Marina Vannucci
marina@rice.edu

## 1 ROI ABBREVIATIONS

ROI abbreviations from case study are shown in Table S1.

## 2 IMPORTANCE-SAMPLING APPROACH TO CROSS-VALIDATION PREDICTION

In this section, we detail the importance-sampling approach to cross-validation prediction of Gelfand (1996). The cross-validation predictive density for the $i$th observation may be written as

$$p(Y_i = 1|\boldsymbol{X}, \boldsymbol{Y}_{-i}) = \int_{\eta,\beta} p(Y_i = 1|\boldsymbol{X}, \boldsymbol{Y}_{-i}, \boldsymbol{\eta}, \boldsymbol{\beta}) p(\boldsymbol{\eta}, \boldsymbol{\beta}|\boldsymbol{X}, \boldsymbol{Y}_{-i}) \partial\boldsymbol{\beta}\partial\boldsymbol{\eta}$$

where we use $p(\boldsymbol{\eta}, \boldsymbol{\beta}|\boldsymbol{X}, \boldsymbol{Y})$ as an importance sampling density for $p(\boldsymbol{\eta}, \boldsymbol{\beta}|\boldsymbol{X}, \boldsymbol{Y}_{-i})$, and $Y_{-i}$ denotes the non-hold out outcomes. More specifically, we estimate the probability that the $i$th observation has outcome $Y_i = 1$ as

$$\mathbb{E}[p(Y_i = 1|\boldsymbol{X}, \boldsymbol{Y}_{-i})] \simeq \frac{\sum_{t=1}^{T} p(Y_i = 1|\boldsymbol{X}_i, \boldsymbol{\eta}^{(t)}, \boldsymbol{\beta}^{(t)}) w^\star(\boldsymbol{\eta}^{(t)}, \boldsymbol{\beta}^{(t)})}{\sum_{t=1}^{T} w^\star(\boldsymbol{\eta}^{(t)}, \boldsymbol{\beta}^{(t)})}, \tag{S1}$$

where the importance weights are given by

$$\begin{aligned} w^\star(\boldsymbol{\eta}^{(t)}, \boldsymbol{\beta}^{(t)}) &= \frac{p(\boldsymbol{\eta}^{(t)}, \boldsymbol{\beta}^{(t)}|\boldsymbol{X}, \boldsymbol{Y}_{-i})}{p(\boldsymbol{\eta}^{(t)}, \boldsymbol{\beta}^{(t)}|\boldsymbol{X}, \boldsymbol{Y})} \\ &\propto \frac{1}{p(Y_i|\boldsymbol{\eta}^{(t)}, \boldsymbol{\beta}^{(t)}, \boldsymbol{X}_i)} \end{aligned}$$

and $\boldsymbol{\eta}^{(t)}$, $\beta^{(t)}$ are the MCMC samples at the $t$th iteration.

| Region | Abbreviation |
|---|---|
| Ipsilateral associative visual cortex | ipsi_AVC |
| Ipsilateral cerebellum | ipsi_Cbm |
| Ipsilateral caudate | ipsi_CN |
| Ipsilateral inf lat pos temporal cortex | ipsi_ipsi_iLPT |
| Ipsilateral inf lat ant temporal cortex | riLAT |
| Ipsilateral inf parietal cortex | ipsi_iPL |
| Ipsilateral lentiform nucleus | ipsi_LN |
| Ipsilateral ant medial temporal cortex | ipsi_MAT |
| Ipsilateral pos medial temporal cortex | ipsi_MPT |
| Ipsilateral pos cingulate cortex | ripsi_PCC |
| Ipsilateral primary visual cortex | ipsi_PVC |
| Ipsilateral sup lat temporal cortex | ipsi_sLT |
| Ipsilateral sup parietal cortex | ipsi_sPL |
| Ipsilateral thalamus | ipsi_Th |
| Ipsilateral parietotemporal cortex | ipsi_PTC |
| Ipsilateral sup frontal cortex | ipsi_GFs |
| Ipsilateral mid frontal cortex | ipsi_GFm |
| Ipsilateral inf frontal cortex | ripsi_Fi |
| Ipsilateral medial frontal cortex | ipsi_GFd |
| Ipsilateral sensorimotor cortex | ipsi_SM |
| Ipsilateral Broca's | ipsi_Broca |
| Ipsilateral ant cingulate cortex | ipsi_GCa |
| Contralateral associative visual cortex | contra_AVC |
| Contralateral cerebellum | contra_Cbm |
| Contralateral caudate | contra_CN |
| Contralateral inf lat pos temporal cortex | contra_iPT |
| Contralateral inf lat ant temporal cortex | contra_iLAT |
| Contralateral inf parietal cortex | contra_iPL |
| Contralateral lentiform nucleus | contra_LN |
| Contralateral ant medial temporal cortex | contra_MAT |
| Contralateral pos medial temporal cortex | contra_MPT |
| Contralateral pos cingulate cortex | contra_PCC |
| Contralateral primary visual cortex | contra_PVC |
| Contralateral sup lat temporal cortex | contra_sLT |
| Contralateral sup parietal cortex | contra_sPL |
| Contralateral thalamus | contra_Th |
| Contralateral parietotemporal cortex | contra_PTC |
| Contralateral sup frontal cortex | contra_GFs |
| Contralateral mid frontal cortex | contra_GFm |
| Contralateral inf frontal cortex | contra_GFi |
| Contralateral medial frontal cortex | contra_GFd |
| Contralateral sensorimotor cortex | contra_SM |
| Contralateral Broca's | contra_Broca |
| Contralateral ant cingulate cortex | contra_GCa |
| Vermis | Vermis |
| Pons | Pons |
| Midbrain | MB |

**Table S1.** Temporal lobe epilepsy dataset: Abbreviations of regions used in case study.

## 3 CONNECTIVITY NETWORK BASED ON RS-FMRI

Rs-fMRI imaging was acquired on a 3T MRI system (Siemens Trio, Erlangen, Germany). Functional imaging was performed with the following parameters: TR=2000 ms, TE=30 ms, FOV=210 mm, matrix=

| Abbreviation | (X,Y,Z) | Abbreviation | (X,Y,Z) |
|---|---|---|---|
| rAVC | (29,20,43) | lAVC | (63,20,43) |
| rCbm | (32,33,23) | lCbm | (58,33,24) |
| rCN | (38,72,42) | lCN | (52,70,42) |
| riLPT | (17,40,29) | liPT | (75,40,29) |
| riLAT | (17,56,23) | liLAT | (75,56,23) |
| riPL | (20,44,63) | liPL | (73,44,63) |
| rLN | (33,63,37) | lLN | (57,63,37) |
| rMAT | (33,55,26) | lMAT | (56,55,26) |
| rMPT | (29,43,26) | lMPT | (61,43,26) |
| rPCC | (42,41,47) | lPCC | (62,43,27) |
| rPVC | (37,17,31) | lPVC | (51,17,31) |
| rsLT | (14,48,44) | lsLT | (76,48,45) |
| rsPL | (26,42,68) | lsPL | (63,42,68) |
| rTh | (39,54,40) | lTh | (51,54,40) |
| rPTC | (20,36,52) | lPTC | (71,36,52) |
| rGFs | (35,81,59) | lGFs | (55,81,59) |
| rGFm | (27,79,53) | lGFm | (62,79,54) |
| rGFi | (22,78,43) | lGFi | (68,78,43) |
| rGFd | (41,88,51) | lGFd | (50,88,51) |
| rSM | (25,54,64) | lSM | (66,54,64) |
| rBroca | (17,72,48) | lBroca | (74,72,48) |
| rGCa | (41,82,45) | lGCa | (48,82,45) |
| Vermis | (45,34,29) | | |
| Pons | (45,52,21) | | |
| MB | (45,51,30) | | |

**Table S2.** Temporal lobe epilepsy dataset: Coordinates of rs-fMRI seeds in standard Montreal Neurological Institute (MNI) space.

$64 \times 64$, slice thickness 4 mm, 34 slices. The imaging sessions included multiple fMRI recordings, each lasting 5 to 15 minutes. For resting state fMRI analysis, 20 minutes of BOLD fMRI data was used for each subject. Preprocessing of rs-fMRI imaging was performed using FSL (fMRIB Software Library) version $5.0.7$ (Oxford, United Kingdom, www.fmrib.ox.ac.uk/fsl) and included head movement artifact correction, non-brain tissue elimination, high-pass filtering ($100$ s), spatial smoothing and mean-based intensity normalization. Tissue-type segmentation was performed on each participant's structural image using FAST (FMRIB's Automated Segmentation Tool) (Zhang et al., 2001), before being aligned to their respective BOLD images. White matter signal and cerebrospinal fluid signals were obtained using the segmented masks. Functional connectivity between the 47 ROIs was estimated by placing a 6-mm spherical seed in Montreal Neurological Institute (MNI) space at the location of each of the 47 ROIs. The coordinates of each sphere in standard MNI space are listed in Table S2 below. Each patient's fMRI BOLD image was registered to the patient's high-resolution structural image using FLIRT (FMRIB's Linear Image Registration Tool) (Jenkinson et al., 2002; Greve and Fischl, 2009), and the high-resolution structural was registered to the standard MNI space using FNIRT (FMRIB's Non-linear Image Registration Tool) (Andersson et al., 2007). The transformation matrix and warpfields were inverted, and then applied to the $47$ spherical seeds to obtain spherical seeds in each individual's BOLD space. Functional connectivity between each pair of nodes was computed as the partial Pearson correlation between the averaged regional time-series. This provided us with a $47 \times 47$ correlation matrix. An edge was then considered as included in the connectivity network if the correlation between the regions exceeded a given threshold. The threshold was chosen so that the average number of neighbors for each region was approximately $5$, yielding a connectivity structure close to a three-dimensional lattice.

# 4 COMPARISON TO PENALIZED REGRESSION METHODS THAT DO NOT CONDITION ON A LATENT STATE

In addition to the comparison to multi-step approaches in Section 3.3, we compared to methods such as elastic net (Zou and Hastie, 2005), ridge regression (Hoerl and Kennard, 1970), and the Least Absolute Shrinkage and Selection Operator (LASSO) method of Tibshirani (1996) that, in particular, do not condition on latent states, but rather use the $\boldsymbol{X}$ data as the covariates. Mixing and regularization parameters for the elastic net using a two-dimensional grid search to minimize cross-validated error. LASSO and ridge regression regularization parameters were chosen through 5-fold cross-validation based on the one standard error rule. Leave-one-out cross validation was used to assess predictive performance. An AUC of 0.64 was obtained with elastic net. Both LASSO and ridge regression failed to predict post-surgical outcome, with all hold-out observations classified as seizure-free.

# 5 PERFORMANCE EVALUATION ON PET-BASED SYNTHETIC DATA

In order to assess our method we evaluate performances using synthetic data that are intended to mimic real PET data. We also investigate comparisons with multi-step approaches as well as approaches that do not condition on latent states.

## 5.1 Synthetic data

We simulated synthetic PET data based on the data we have available from the University of California, Los Angeles Seizure Disorder Center. Our approach is similar in spirit to the simulation strategies adopted by Zhang et al. (2014) and Quirós et al. (2010) for fMRI data. Specifically, we considered real PET data from a single patient and obtained synthetic data on $n = 20$ subjects as

$$\boldsymbol{X}_{syn,i} = \boldsymbol{X} + \boldsymbol{\delta}_i,$$

where $\boldsymbol{X}$ denotes the PET data from the selected patient from the real PET study, and $\boldsymbol{\delta}_i$ is simulated as described below. We generated data for $K = 2$ groups of subjects, with $n_1 = 10$ subjects in group 1 and $n_2 = 10$ subjects in group 2. For patients in group 1, we set $\boldsymbol{\delta}_i$ to be an $R$-dimensional vector with $\delta_{i,j} \sim \text{Unif}(0.5, 1)$ for all $\gamma_j = 0$ and $\delta_{i,j} \sim \text{Unif}(0.4, 0.7)$ for all $\gamma_j = 1$. For patients in group 2, we set $\boldsymbol{\delta}_i$ to be an $R$-dimensional vector with $\delta_{i,j} \sim \text{Unif}(0.5, 1)$ for $\gamma_j = 0$ and $\delta_{i,j} \sim \text{Unif}(0.8, 1.1)$ for $\gamma_j = 1$. The true map of $\boldsymbol{\gamma}$ was set to define 6 ROIs with $\gamma_j = 1$, located in the bilateral caudate, bilateral lentiform nuclei, and bilateral primary visual cortices, as shown in Figure S1. In order to simulate the clinical outcome data, we set

$$Y_i \quad \sim \quad \text{Bern}\left(\frac{e^{\boldsymbol{\xi}_i^T \boldsymbol{\beta}}}{1 + e^{\boldsymbol{\xi}_i^T \boldsymbol{\beta}}}\right), \tag{S2}$$

with $\boldsymbol{\xi}_i = (1, I(\eta_i = 1))$ and $\boldsymbol{\beta} = (2.3, -4.5)$. Our final synthetic dataset comprised data on $R = 47$ regions for $n = 20$ subjects. Data were column-centered prior to the analysis.

### 5.1.1 Results

As done in the case study, we set hyperparameters to be weakly informative. We set the unscaled variance of the ICAR prior to $c_k = 5$, for $k = 1, 2$. We placed a vague prior on the hyperparameters of the mixing proportions $\boldsymbol{\pi}$, that is, $\alpha_k = 1$, and fixed the prior shape and scale parameters of the inverse gamma priors

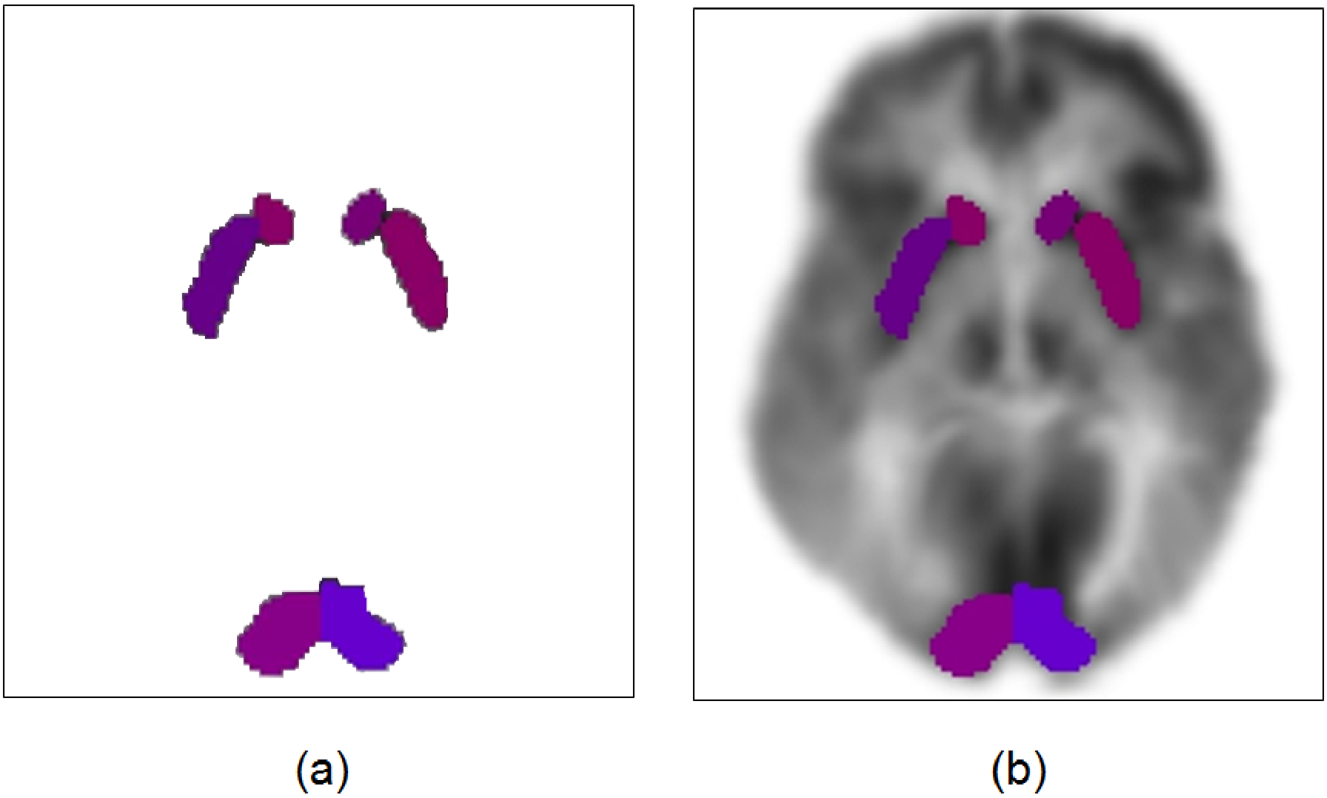


Figure S1: Synthetic data: (a) True map of $\boldsymbol{\gamma}$, with $\gamma_j = 1$ for bilateral caudate, bilateral lentiform nuclei, and primary visual cortices; (b) Overlay of true map of $\gamma$ on slice 36 of PET image.

on $\sigma_k$ and $\sigma_0$ to be non-informative with $a_0 = 2$, $b_0 = 1$, $a_k = 2$, and $b_k = 1$. We also fixed the prior mean and covariance of $\boldsymbol{\beta}$ to $\boldsymbol{m}_\beta = \mathbf{0}$ and $V_\beta = 5 \cdot \mathbb{I}_K$, respectively. We set the neighborhood matrix, $S$, of the MRF prior and the ICAR prior to a binary matrix with the bilateral caudate, bilateral lentiform nuclei, bilateral primary visual cortices, bilateral associative visual cortices, ipsilateral thalamus, and contralateral Broca's area as neighbors. We first show results we obtained by setting the MRF parameters to $e = -1.4$, which implies a lower bound on the prior probability of selection of 20% of the total number of regions, and $f = 0.1$, and then comment on sensitivity below.

When running the MCMC, we initialized the chain with $0.5R$ randomly selected regions as discriminatory and an equal number of randomly selected observations assigned to the two clusters. We initialized the remaining values as $\boldsymbol{\mu}_k^{(0)} = \mathbf{0}$, $\pi_k^{(0)} = 1/K$, $\sigma_k^{(0)} = 1$, for $k = 1, 2$, and $\sigma_0^{(0)} = 1$, $\beta^{(0)} = \mathbf{0}$. We ran the MCMC sampler for 20,000 iterations, with the first 10,000 sweeps discarded as burn-in. The Geweke z-statistic ranged from 0.61-1.14, failing to reject the null of equality between the means of the first 10% and last 50% of the chain. The Raftery-Lewis dependence factor, calculated on the sampled values of $\boldsymbol{\beta}$, ranged from 1.19-1.26, indicating low autocorrelation of the chain. With our R implementation, 1,000 iterations of the MCMC algorithm ran in 33.9s on an Intel Core i7 station (2.50 GHz) with 16GB RAM.

Figure S2 shows the marginal posterior probabilities of inclusion (PPIs) for each of the $47$ brain regions. The median model here results in the accurate identification of the 6 discriminatory regions. Also, a classification of the subjects into two subgroups, based on the posterior mode of class allocation, led to accurately classify all subjects to the correct subgroups. Finally, the posterior means and 95% credible

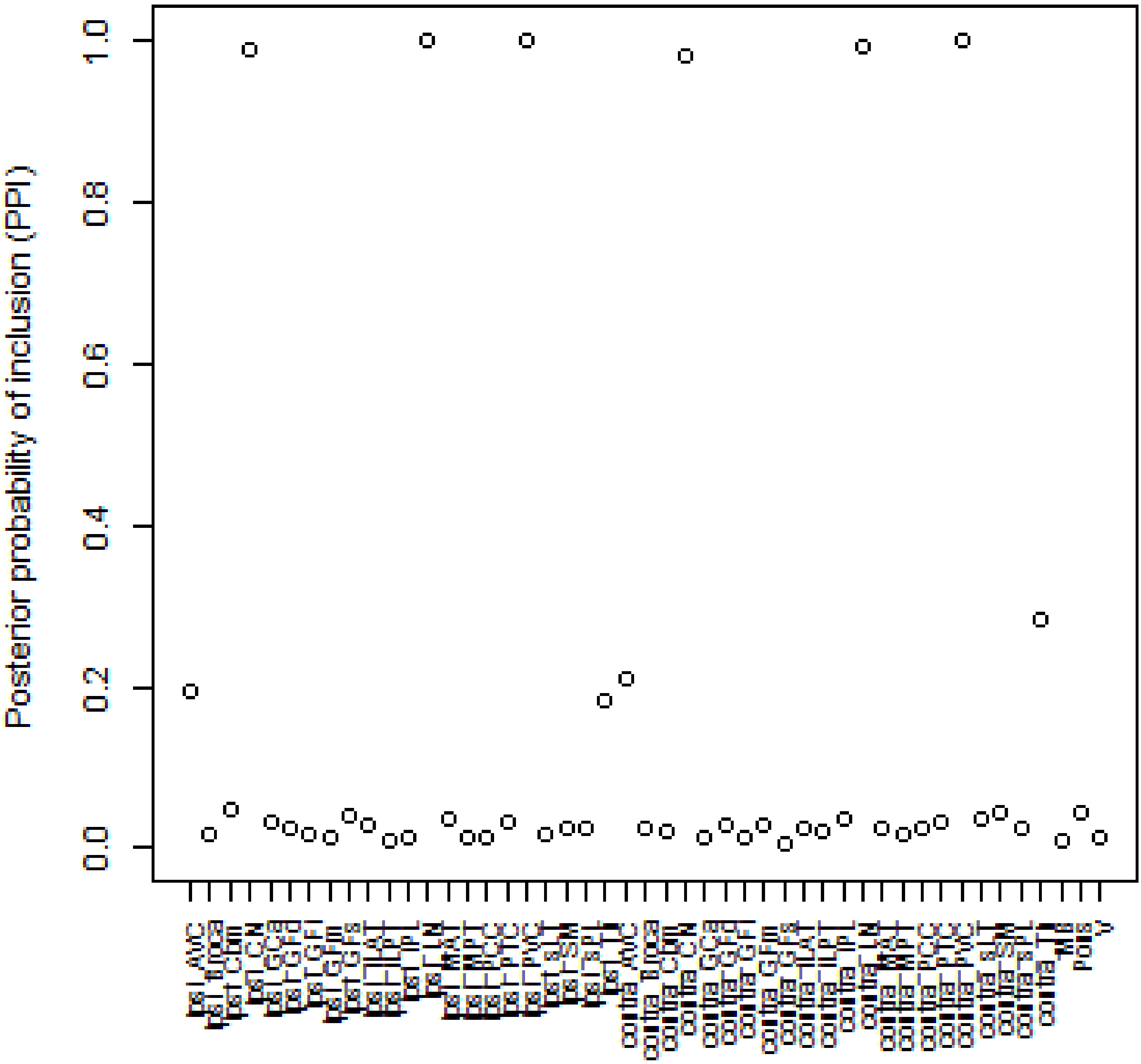


Figure S2: Synthetic data: Marginal posterior probability of inclusion for each of the $47$ brain regions.

intervals for $\beta_0$ and $\beta_1$ were estimated as 2.82 (1.13,4.88) and -4.26 (-6.46,-2.28), respectively, leading to good estimates of these parameters, with true values contained in the 95% credible intervals.

In order to better assess the selection performances of our method, we looked at results over repeated simulations and calculated FPR (false positive rate), FNR (false negative rate), accuracy and $F_1$-score, all averaged over 30 replicated datasets. Here, FPR is defined as $FPR = \frac{FP}{FP+TN}$, with $FP$ the number of false positives and $TN$ the number of true negatives. FNR is defined as $FNR = \frac{FN}{FN+TP}$, with $FN$ the

| | | $e$ | | | |
|---|---|---|---|---|---|
| | | -4.5 | -3 | -2.2 | -1.4 |
| $\gamma$ | FPR | 0.000 | 0.000 | 0.000 | 0.000 |
| | FNR | 0.311 | 0.067 | 0.033 | 0.030 |
| | Accuracy | 0.960 | 0.991 | 0.996 | 0.996 |
| | $F_1$-score | 0.693 | 0.933 | 0.967 | 0.967 |
| $\eta$ | Rand Index | 0.91 | 0.96 | 0.98 | 0.98 |
| $\beta_0$ | Mean (SD) | 0.62 (1.11) | 1.82 (0.89) | 1.91 (0.83) | 1.91 (0.81) |
| $\beta_1$ | Mean (SD) | -1.29 (2.28) | -3.58 (1.40) | -3.70 (1.17) | -3.67 (1.16) |

**Table S3.** Synthetic data: Sensitivity analysis to the MRF hyperparameter $e$, for fixed $f = 0.1$. Results are averaged over 30 replicated datasets.

number of false negatives and $TP$ the number of true positives. Accuracy is defined as

$$Accuracy \quad = \quad \frac{TP + TN}{TP + TN + FP + FN}.$$

Lastly, $F_1$-score is defined as

$$F_1 = 2 \cdot \frac{(TP/(TP + FP))\,(TP/(TP + FN))}{(TP/(TP + FP)) + (TP/(TP + FN))}.$$

With the hyperparameters setting described above, we obtained FPR= 0.000, FNR=0.030, accuracy of 0.996 and an $F_1$-score of 0.967. For cluster allocation, we quantified performances via the Rand index (RI) of (Rand, 1971). Let us define the following quantities: $A = \sum_{i>i'} I(z_i = z_{i'})I(\hat{z}_i = \hat{z}_{i'})$, the number of pairs of observations that belong to the same group in both the true clustering and the estimated one; $B = \sum_{i>i'} I(z_i = z_{i'})I(\hat{z}_i \neq \hat{z}_{i'})$, the number of pairs in the same group in the true clustering and in different groups in the estimated one; $C = \sum_{i>i'} I(z_i \neq z_{i'})I(\hat{z}_i = \hat{z}_{i'})$, the number of pairs in different groups in both the true and estimated clustering; $D = \sum_{i>i'} I(z_i \neq z_{i'})I(\hat{z}_i \neq \hat{z}_{i'})$, the number of pairs in different groups in both truth and estimate. Then the RI is defined as

$$RI = \frac{A + D}{A + B + C + D},$$

and takes values between 0 and 1. The larger the index, the more accurate the clustering result. We obtained a mean Rand index of $RI = 0.98$, over the 30 replicates. Furthermore, the means and standard deviations of the posterior mean estimates for $\beta_0$ and $\beta_1$ were 1.91 (0.81) and -3.67 (1.16), respectively.

When investigating sensitivity to the prior choices, we noticed that modest changes of the values of the model parameters did not affect the accuracy of the estimation results while, as expected, we observed some sensitivity of the selection results to the parameters of the Markov random field prior, $e$ and $f$. Tables S3-S4 report results on FPR, FNR, accuracy, $F_1$-score, Rand index and estimated $\beta$'s, all calculated over 30 replicates, for several choices of these parameters. Larger values of $e$ lead to lower FNRs, while larger values of $f$ lead to higher FPRs and lower precisions. Also, as expected, lower accuracy of variable selection was associated with a lower Rand index for cluster allocation and larger errors in the estimation of $\beta_0$ and $\beta_1$.

## 5.2 Comparison study

For comparison, we first assessed the performance of our unified method, which performs simultaneous clustering based on selected variables and outcome prediction, against multi-step approaches that focus

| | | $f$ | | | | |
|---|---|---|---|---|---|---|
| | | 0.1 | 0.2 | 0.3 | 0.5 | 0.7 |
| $\gamma$ | FPR | 0.000 | 0.018 | 0.091 | 0.097 | 0.098 |
| | FNR | 0.030 | 0.067 | 0.067 | 0.000 | 0.000 |
| | Accuracy | 0.996 | 0.976 | 0.912 | 0.914 | 0.915 |
| | $F_1$-score | 0.967 | 0.879 | 0.700 | 0.750 | 0.750 |
| $\eta$ | Rand Index | 0.98 | 0.96 | 0.96 | 0.51 | 0.53 |
| $\beta_0$ | Mean (SD) | 1.91 (0.81) | 1.87 (0.89) | 1.84 (0.85) | 0.10 (0.44) | 0.15 (0.59) |
| $\beta_1$ | Mean (SD) | -3.67 (1.16) | -3.62 (1.32) | -3.65 (1.29) | -0.25 (1.00) | -0.36 (1.22) |

**Table S4.** Synthetic data: Sensitivity analysis to the MRF hyperparameter $f$, for fixed $e = -1.4$. Results are averaged over 30 replicated datasets.

| | | Proposed | Multi-step methods | | |
|---|---|---|---|---|---|
| | | | LASSO | Elastic net | Logistic reg |
| $\gamma$ | FPR | 0.000 | 0.037 | 0.085 | 0.094 |
| | FNR | 0.030 | 0.550 | 0.089 | 0.000 |
| | Accuracy | 0.996 | 0.897 | 0.914 | 0.918 |
| | $F_1$-score | 0.967 | 0.516 | 0.784 | 0.754 |
| $\eta$ | Rand Index | 0.98 | 0.96 | 0.95 | 1.00 |
| $\beta_0$ | Mean (SD) | 1.91 (0.81) | 12.35 (11.23) | 12.34 (11.22) | 12.44 (11.13) |
| $\beta_1$ | Mean (SD) | -3.67 (1.16) | -23.52 (19.12) | -23.52 (19.11) | 23.72 (18.86) |

**Table S5.** Synthetic data: Performance comparison between the proposed method and multi-step approaches. Results are averaged over 30 replicated datasets.

solely on clustering or solely on outcome prediction. We considered three multi-step procedures. In the first two approaches, regularized logistic regression through either (a) the Least Absolute Shrinkage and Selection Operator (LASSO) method of Tibshirani (1996) or the (b) elastic net penalized regression (Zou and Hastie, 2005) was first used to perform variable selection, using the observed outcome $Y$ as the dependent variable. The LASSO regularization parameter, $\lambda$, was chosen to minimize the 5-fold cross-validation error. Regularization and mixing parameters in the elastic net were optimized using a two-dimensional grid search to minimize cross-validated error. Imaging data were standardized by centering and scaling prior to penalized regression, as suggested by Efron et al. (2004). A Gaussian mixture model was then fitted to the selected regions using the expectation-maximization (EM) algorithm to obtain estimates of the cluster memberships $\boldsymbol{\eta}$ (Fraley and Raftery, 2006). These estimates of $\boldsymbol{\eta}$ were then used as the covariates in a logistic regression. The third multi-step approach we considered mimics an approach in which a subset of regions is selected a priori and used in a subsequent two-stage procedure. In this approach, for each subject, permutation $p$-values were calculated for each region and then the subject-level $p$-value maps were combined into a group-level $p$-value map using Fisher's method (Fisher, 1950). A subset of the regions was selected by thresholding the group-level $p$-value map with family-wise error rate control at the $0.05$ level. Then, a $k$-means cluster analysis was performed using the selected regions to obtain estimates of $\boldsymbol{\eta}$. Finally, a logistic regression was fitted using the estimates of $\boldsymbol{\eta}$ as the covariates in a logistic regression.

For all methods, we report results averaged over 30 replicated datasets. We evaluated variable selection performance via FNR, FPR, accuracy and $F_1$-score, and cluster allocation performance via the Rand index. Results are reported in Table S5. Lower accuracy of selection is observed for all three multi-step methods. Whereas the LASSO multi-step approach tends to underselect brain regions (FNR, 55.0%), the elastic net and logistic regression approaches tend to overselect (FPR, 8.5% and 9.4%, respectively). As for the estimation of $\boldsymbol{\beta}$, our unified method achieves estimates of $\boldsymbol{\beta}$ with higher accuracy as well as lower variance than all three multi-step methods. These results demonstrate that a unified and probabilistically coherent modeling approach can achieve improved estimation performance with respect to multi-step approaches.

| | Proposed | Regularized methods (no latent state) | |
|---|---|---|---|
| | | LASSO | Elastic net |
| AUC, Mean (SD) | 0.91 (0.11) | 0.87 (0.15) | 0.88 (0.13) |
| Cross-entropy, Mean (SD) | 0.35 (0.16) | 0.47 (0.28) | 0.48 (0.12) |

**Table S6.** Synthetic data: Comparison of predictive performance between the proposed method and approaches which do not condition on latent states. Results are averaged over 30 replicated datasets.

Next, we demonstrate that, when predictors are characterized by a true latent structure, as in our simulated scenario, a modeling approach that explicitly accounts for such structure is able to achieve superior prediction performance when compared to approaches that do not properly account for such heterogeneity in the data. As done in the case study, we focus the comparison on elastic net and LASSO, which do not condition on latent states but rather use the $\mathbf{X}$ data as the covariates. However, instead of calculating cross-validation predictions, for this comparison we simulated an additional set of $n_{test} = 15$ synthetic observations, that comprised our validation set, and calculated posterior predictive probabilities for our method as described in Section 2.3.5. As above, regularization and mixing parameters for elastic net were optimized using a two-dimensional grid search to minimize cross-validated error. LASSO and ridge regularization parameters were chosen to minimize 5-fold cross-validation error. Predictive accuracy was assessed for all methods through hold-out validation on the set of $n_{test} = 15$ synthetic observations. We calculated two measures of predictive performance: the AUC and the cross-entropy error measure, defined as

$$\text{Cross-entropy} = \frac{1}{n_{test}} \sum_{i=1}^{n_{test}} [Y_i \log \hat{Y}_i + (1 - Y_i) \log(1 - \hat{Y}_i)],$$

where $\hat{Y}_i$ is the predicted outcome for subject $i$. Mean AUC and cross-entropy error, averaged over 30 replicates, are reported in Table S6 and show that our method attains both lower cross-entropy as well as higher AUC compared to the competing regularized techniques.

# 6 MCMC ALGORITHM FOR POSTERIOR INFERENCE

A generic iteration of the MCMC algorithm comprises the following steps:

1. **Update $\sigma_k$ and $\sigma_0$:** These are Gibbs steps, $\sigma_k \sim \text{IG}(\sigma_k^{(a)}, \sigma_k^{(b)})$ for all $k$, and $\sigma_0 \sim \text{IG}(\sigma_0^{(a)}, \sigma_0^{(b)})$, as given in the main paper.
2. **Update $\pi$:** This is a Gibbs step, with $\pi \sim \text{Dirichlet}(\alpha_1 + n_1, \alpha_2 + n_2, \ldots, \alpha_K + n_K)$.
3. **Jointly update $(\boldsymbol{\gamma}, \{\boldsymbol{\mu}_k\}_{k=1}^K)$:** This is a joint Metropolis-Hastings step. To propose a new candidate $\boldsymbol{\gamma}^\star$, randomly choose between two transition moves:
   a. Add/delete: Randomly choose one of the $R$ indices in $\boldsymbol{\gamma}$, and change its value either from 0 to 1, or 1 to 0.
   b. Swap: Choose independently and at random a 0 and 1 in $\boldsymbol{\gamma}$, and switch their values.

   For all $\mu_{k,j}$'s such that $\gamma_j^\star = 1$, sample $\mu_{k,j}$ from its full conditional, i.e. $\mu_{k,j}^\star \sim \text{N}(\zeta_\mu, \tau_\mu)$, $k = 1, \ldots K$, with $\zeta_\mu$ and $\tau_\mu$ as given in the main paper. The proposed candidate $\left(\boldsymbol{\gamma}^\star, \{\boldsymbol{\mu}_k^\star\}_{k=1}^K\right)$ is jointly

| | $f = 0.01$ | $f = 0.1$ |
|---|---|---|
| Geweke z-statistic ($\lvert z_G \rvert$) | 0.48-2.03 | 0.07-1.82 |
| Raftery-Lewis dependence factor | 1.15-4.32 | 1.14-3.71 |
| Gelman-Rubin PSRF | 1.00-1.00 | 1.00-1.13 |

**Table S7.** Temporal lobe epilepsy dataset: Markov chain convergence tests.

accepted with probability

$$\begin{aligned} & \min\left\{1, \frac{p(\boldsymbol{\gamma}^\star, \boldsymbol{\mu}_1^\star, \ldots, \boldsymbol{\mu}_K^\star | X, \boldsymbol{\eta}, \Sigma_1, \ldots, \Sigma_K)}{p(\boldsymbol{\gamma}, \boldsymbol{\mu}_1, \ldots, \boldsymbol{\mu}_K | X, \boldsymbol{\eta}, \Sigma_1, \ldots, \Sigma_K)}\right\} \\ \approx \quad & \min\left\{1, \frac{L(X|\boldsymbol{\eta}, \boldsymbol{\mu}_1^\star, \ldots, \boldsymbol{\mu}_K^\star, \Sigma_1, \ldots, \Sigma_K)\left[\prod_{k=1}^K p(\boldsymbol{\mu}_k^\star|\boldsymbol{\gamma}^\star)\right] p(\boldsymbol{\gamma}^\star)}{L(X|\boldsymbol{\eta}, \boldsymbol{\mu}_1, \ldots, \boldsymbol{\mu}_K, \Sigma_1, \ldots, \Sigma_K)\left[\prod_{k=1}^K p(\boldsymbol{\mu}_k|\boldsymbol{\gamma})\right] p(\boldsymbol{\gamma})}\right\} \end{aligned}$$

where $p(\boldsymbol{\mu}_k|\boldsymbol{\gamma}) = \prod_{j=1}^R p(\mu_{k,j}|\gamma_j)\ \forall k = 1, \ldots, K$, and $p(\boldsymbol{\gamma}) \propto \exp\left\{e\mathbf{1}_R^T\boldsymbol{\gamma} + f\boldsymbol{\gamma}^T S\boldsymbol{\gamma}\right\}$.

**Resampling step on** $\{\boldsymbol{\mu}_{k(\gamma)}\}_{k=1}^K$: At every $M$th sweep, for all $\mu_{k,j}$'s such that $\gamma_j = 1$, sample $\mu_{k,j} \sim \mathrm{N}(\zeta_\mu, \tau_\mu)$, $k = 1, \ldots K$. This step is done only to improve the mixing of the chain.

4. **Update** $\boldsymbol{\beta}$: This is a Gibbs step, $\boldsymbol{\beta} \sim \mathrm{N}(\zeta_\beta, \tau_\beta)$.
5. **Update** $\omega$: This is a Gibbs step, $\omega_i \sim \mathrm{PG}(1, \boldsymbol{\xi}_i^T\boldsymbol{\beta}), i = 1, \ldots, n$.
6. **Update** $\boldsymbol{\eta}$: This is a Gibbs step,
$p(\eta_i|\cdot) \propto \pi_k p(\boldsymbol{X}_{i(\boldsymbol{\gamma})}|\eta_i = k, \boldsymbol{\mu}_k, \Sigma_k)p(Y_i|\eta_i = k, \boldsymbol{\beta}, \omega_i), i = 1, \ldots, n$.

An identifiability problem arises in finite mixture models due to the invariance of the likelihood under $K!$ permutations of the component labels, resulting in equal posterior values under each permutation. This issue is usually referred to as "label-switching" and leads to problems with estimating component-specific quantities. In order to account for this, we adapted the post-MCMC decision-theoretic relabeling method of Stephens (2000) to our modeling framework. This method defines an appropriate loss function based on the Kullback-Leibler divergence, and postprocesses the MCMC output to minimize the posterior expected loss.

## 7 CONVERGENCE TESTS

Convergence of each individual chain was assessed using the Raftery-Lewis diagnostic (Raftery et al., 1992) and the Geweke test (Geweke et al., 1991). Convergence of the multiple chains was assessed using the Gelman-Rubin potential scale reduction factor, based on the implementation in the R package "coda" (Raftery and Lewis, 1992). Values of the diagnostics tests for the results reported in the paper are given in Table S7 and indicate convergence to the stationary distribution.

## CONFLICT OF INTEREST STATEMENT

The authors declare that the research was conducted in the absence of any commercial or financial relationships that could be construed as a potential conflict of interest.

## AUTHOR CONTRIBUTIONS

SC, MG, ZH, JS and MV contributed to the design and analysis of the work; HY, SD and JS contributed to the data acquisition; SC, MG and MV wrote the paper; all authors revised the manuscript critically for important intellectual content.

## FUNDING

Marina Vannucci and Michele Guindani are partially supported by NSF SES-1659925 and NSF SES-1659921. Sharon Chiang was supported by the National Library of Medicine Training Fellowship in Biomedical Informatics, Gulf Coast Consortia for Quantitative Biomedical Sciences (Grant #2T15-LM007093-21) and by the National Institute of Health (Grant #5T32-CA096520-07). Zulfi Haneef is partially supported by The Epilepsy Foundation of America (Award ID 244976), the Baylor College of Medicine, Computational and Integrative Biomedical Research Center (CIBR) Seed Grant Awards and the Baylor College of Medicine Junior Faculty Seed Funding Program Grant. John M. Stern is partially supported by NIH-NINDS K23 Grant NS044936 and The Leff Family Foundation.

## ACKNOWLEDGMENTS

The authors would like to give special thanks to Daniel H.S. Silverman, Stefan T. Nguyen, Navya M. Reddy, and Regina Ahn (University of California, Los Angeles) for provision of the NeuroQ software, pre-processing of PET data, and organization support. The authors also express their grateful appreciation to Wesley Kerr (University of California, Los Angeles) for data management of the PET records and helpful insights.

## REFERENCES

Andersson, J. L., Jenkinson, M., Smith, S., et al. (2007). *Non-linear registration, aka Spatial normalisation FMRIB technical report TR07JA2*. Tech. rep., FMRIB Centre

Efron, B., Hastie, T., Johnstone, I., Tibshirani, R., et al. (2004). Least angle regression. *The Annals of Statistics* 32, 407–499

Fisher, R. A. (1950). *Statistical methods for research workers* (London: Oliver and Boyd), 11 edn.

Fraley, C. and Raftery, A. E. (2006). *MCLUST version 3: an R package for normal mixture modeling and model-based clustering*. Tech. Rep. 504, Department of Statistics, University of Washington, Seattle

Gelfand, A. E. (1996). Model determination using sampling-based methods. In *Markov Chain Monte Carlo in Practice*, eds. W. R. Gilks, S. Richardson, and D. Spiegelhalter (London: Chapman and Hall), chap. 9. 145–161

Geweke, J. et al. (1991). *Evaluating the accuracy of sampling-based approaches to the calculation of posterior moments*, vol. 196 (Federal Reserve Bank of Minneapolis, Research Department Minneapolis, MN, USA)

Greve, D. N. and Fischl, B. (2009). Accurate and robust brain image alignment using boundary-based registration. *NeuroImage* 48, 63–72

Hoerl, A. E. and Kennard, R. W. (1970). Ridge regression: Biased estimation for nonorthogonal problems. *Technometrics* 12, 55–67

Jenkinson, M., Bannister, P., Brady, M., and Smith, S. (2002). Improved optimization for the robust and accurate linear registration and motion correction of brain images. *NeuroImage* 17, 825–841

Quirós, A., Diez, R. M., and Gamerman, D. (2010). Bayesian spatiotemporal model of fMRI data. *NeuroImage* 49, 442–456

Raftery, A. E., Lewis, S., et al. (1992). How many iterations in the Gibbs sampler. *Bayesian Statistics* 4, 763–773

Raftery, A. E. and Lewis, S. M. (1992). One long run with diagnostics: Implementation strategies for Markov chain Monte Carlo. *Statistical Science* 7, 493–497

Rand, W. M. (1971). Objective criteria for the evaluation of clustering methods. *Journal of the American Statistical association* 66, 846–850

Stephens, M. (2000). Dealing with label switching in mixture models. *Journal of the Royal Statistical Society: Series B (Statistical Methodology)* 62, 795–809

Tibshirani, R. (1996). Regression shrinkage and selection via the lasso. *Journal of the Royal Statistical Society. Series B (Methodological)* 58, 267–288

Zhang, L., Guindani, M., Versace, F., and Vannucci, M. (2014). A spatio-temporal nonparametric Bayesian variable selection model of fMRI data for clustering correlated time courses. *NeuroImage* 95, 162–175

Zhang, Y., Brady, M., and Smith, S. (2001). Segmentation of brain MR images through a hidden Markov random field model and the expectation-maximization algorithm. *IEEE Transactions on Medical Imaging* 20, 45–57

Zou, H. and Hastie, T. (2005). Regularization and variable selection via the elastic net. *Journal of the Royal Statistical Society: Series B (Statistical Methodology)* 67, 301–320
